# Systematic analysis of off-target effects in an RNAi screen reveals microRNAs affecting sensitivity to TRAIL-induced apoptosis

**DOI:** 10.1186/1471-2164-11-175

**Published:** 2010-03-15

**Authors:** Ian Sudbery, Anton J Enright, Andrew G Fraser, Ian Dunham

**Affiliations:** 1 Work performed at: Wellcome Trust Sanger Institute, The Wellcome Trust Genome Campus, Hinxton, Cambridge, UK; 2 Department of Systems Biology, Harvard Medical School, Boston, USA; 3 European Bioinformatics Institute, The Wellcome Trust Genome Campus, Hinxton, Cambridge, UK; 4 Terrence Donnelly Centre for Cellular and Biomolecular Research, 160 College Street, Toronto, Ontario, Canada

## Abstract

**Background:**

RNA inhibition by siRNAs is a frequently used approach to identify genes required for specific biological processes. However RNAi screening using siRNAs is hampered by non-specific or off target effects of the siRNAs, making it difficult to separate genuine hits from false positives. It is thought that many of the off-target effects seen in RNAi experiments are due to siRNAs acting as microRNAs (miRNAs), causing a reduction in gene expression of unintended targets via matches to the 6 or 7 nt 'seed' sequence. We have conducted a careful examination of off-target effects during an siRNA screen for novel regulators of the TRAIL apoptosis induction pathway(s).

**Results:**

We identified 3 hexamers and 3 heptamer seed sequences that appeared multiple times in the top twenty siRNAs in the TRAIL apoptosis screen. Using a novel statistical enrichment approach, we systematically identified a further 17 hexamer and 13 heptamer seed sequences enriched in high scoring siRNAs. The presence of one of these seeds sequences (which could explain 6 of 8 confirmed off-target effects) is sufficient to elicit a phenotype. Three of these seed sequences appear in the human miRNAs miR-26a, miR-145 and miR-384. Transfection of mimics of these miRNAs protects several cell types from TRAIL-induced cell death.

**Conclusions:**

We have demonstrated a role for miR-26a, miR-145 and miR-26a in TRAIL-induced apoptosis. Further these results show that RNAi screening enriches for siRNAs with relevant off-target effects. Some of these effects can be identified by the over-representation of certain seed sequences in high-scoring siRNAs and we demonstrate the usefulness of such systematic analysis of enriched seed sequences.

## Background

TNF-related apoptosis inducing ligand (TRAIL) induces apoptosis in many transformed cell types but not in most normal cell types [1,2]. This makes it an attractive option for anti-cancer treatment. Indeed several clinical trials are underway using either recombinant ligand or antibodies targeted at the TRAIL receptors [3]. However, not all tumour cells are sensitive to TRAIL and the mechanism underlying resistance to TRAIL killing is not fully understood.

TRAIL stimulates apoptosis by binding to one of two receptors, TNFRSF10A and TNFRSF10B [4,5]. This leads to the recruitment of FADD and Caspase-8, which leads, in turn, both to the direct activation of the executioner caspase, Caspase-3, and to the activation of the mitochondrial apoptosis pathway via the cleavage of BID [6,7]. Mitochondrial pathway activation results both in the activation of Caspase-3 by Caspase-9 and also relief of Caspase-3 inhibition by the protein DIABLO [8].

Investigations into the mechanisms that regulate sensitivity of cells to TRAIL have implicated many factors and pathways. Regulation of the TRAIL receptors, at the level of expression, localisation to the cell surface and the O-glycosylation of the proteins, partially, but not fully correlates with sensitivity [9-11]. Levels of the CFLAR (also known as cFLIP) apoptosis inhibitory factor has also been associated with TRAIL resistance, although there is not a correlation in all cases [9,10]. Other factors that have been implicated include MYC, RAS, Protein Kinase C, ATK and IGF1 [12-20].

Genome scale gene knock-down screens using RNA interference (RNAi) are an increasingly popular method for finding genes associated with cellular phenotypes. Indeed two screens for genes involved in TRAIL-induced apoptosis have been undertaken [17,21]. Despite screening many of the same genes, only a single gene was identified in both screens. This could be an indication that these screens were not saturating, that there are cell-type specific differences, or that the confirmation procedures/control of off-target effects were insufficient in one or both screens.

Off-target effects in RNAi screens occur where short interfering RNAs (siRNAs) directly affect the expression of genes other than the one that they are designed to target. Micro-array experiments have shown that an siRNA can affect the mRNA levels of many genes [22,23]. There is growing evidence that at least some of these effects are due to siRNAs acting as microRNAs (miRNAs), targeting transcripts which contain matches in their 3' untranslated regions (UTRs) to 6 or 7 nucleotide sequences at the 5' end of the siRNAs (nucleotides 2 to 7 or 8, the seed sequence). Firstly the transcripts shown to be reduced after siRNA transfection in microarray experiments correlate with the presence and number of matches to the seed sequence of the transfected siRNAs, but not with overall identity between the siRNA and the best match in an mRNA [22]. Secondly, the degradation products of mRNAs with an imperfect match to an siRNA do not correlate with the canonical cleavage site associated with RNAi induced degradation, but are more random, as in miRNA-induced de-stabilisation of mRNA [24]. Finally, siRNAs can affect the protein levels of mis-matched targets, without affecting the mRNA levels, particularly if GU base pairing is present [24,25].

Such off-target effects have been shown to cause problems in siRNA based screens. In a screen for genes which sensitize normally resistant cells to a Bcl-2 inhibitor, Lin *et al *found that their top three hits scored well due to off-target effects. They observed that two of these three siRNAs shared the same 7 nt seed sequence. Searching a database of 3' UTRs, they found that the *Bcl-2 *family member *Mcl-1 *contained matches to both the 7 nt seed sequences seen in their top three siRNAs. They hypothesised that screening enriches for siRNAs with off-target effects and that this will lead to enrichment of certain seed sequence in high scoring siRNAs [26].

Until now, such effects in siRNA screens have only been described as post-hoc explanations for already identified off-target effects, generally restricted to the seeds of the top few siRNAs from a hit list. To the best of our knowledge, systematic analyses of the effects of seed sequences on siRNA screen results have yet to be reported. Therefore, here we report a screen for regulators of the TRAIL-induced apoptosis pathway. After rigorous confirmation of a set of hits, we investigated the off-target effects in our results. We show both that a set of seed sequences is over-represented in our potential hit siRNAs and that a second, overlapping set of seed sequences is enriched in high scoring siRNAs in general and that the presence of one of these seed sequences is sufficient to render an siRNA active in our screen. This effect was used to identify three species of miRNA that are regulators of the TRAIL induced apoptosis pathway.

## Results and Discussion

### A screen for novel regulators of the TRAIL-induced pathway

**Figure 1:**
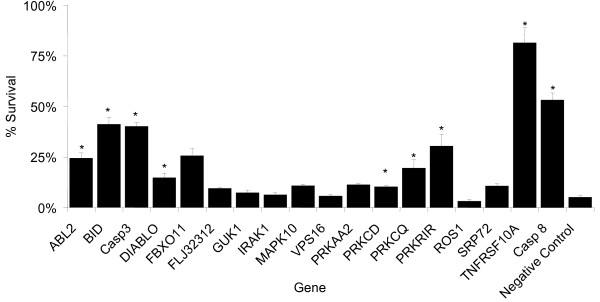
**Knock-down of many genes previously associated with the TRAIL pathway reduces sensitivity to TRAIL-induced cell death**. HeLa Cells were transfected with siRNAs targeting a selection of genes previously associated with TRAIL induced apoptosis. After 48 hrs the sensitivity of cells to treatment with 1 μg/ml TRAIL was measured (see Materials and Methods). Error bars represent 1 standard deviation (n = 3), * indicates significantly different from negative control (t-test on log-transformed data, Bonferroni correct p < 0.05).

In order to confirm that our assay is capable of identifying novel genes in the TRAIL-induced apoptosis pathway we selected 18 genes previously implicated in the pathway, including several genes identified in a previous screen, in addition to genes central to the apoptosis pathway [17]. We measured the effect of transfecting siRNAs targeting these genes on the sensitivity of HeLa cells to TRAIL-induced cell death. 50% of these siRNAs significantly reduced the sensitivity of cells to TRAIL-induced cell death, including 5/5 core death pathway genes (*BID, CASP3, CASP8, DIABLO and TNFRSF10A*, Figure 1). Comparing the TRAIL sensitivity of cells transfected with a negative control siRNA to cells transfected with an anti-CASP8 siRNA, shows that the assay had a Z' score of 0.46 (data not shown). Z' is a scale-free measurement of the separation of two distributions where scores of greater than zero indicate non-overlapping distributions, while a score of one would indicate infinite separation (either zero standard deviation for each distribution or an infinite separation of the means) [27]. This demonstrates the effectiveness of our RNAi assay for identifying genes in the TRAIL pathway.

**Figure 2:**
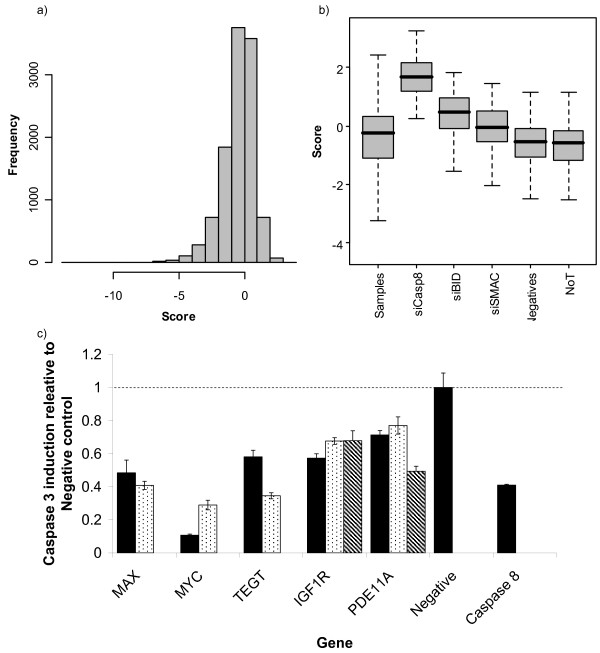
**A screen for new modulators of the TRAIL-induced apoptosis pathway**. 12,190 siRNAs targeting 6,095 genes were screened in duplicate for genes affecting sensitivity to TRAIL induced pathway (see materials and methods). a) Distribution of scores for sample siRNAs. Survival after treatment was median normalised by plate, a z-like score calculated using median and median absolute deviation and the minimum of two replicates was selected for each siRNA (see methods and [45]). This score represents a normalized measure of the effect of a particular siRNA compared to the majority of siRNAs and is robust to outliers. b) Boxplot of scores for each of groups of controls used in the screen. NoT = Not Transfected c) Induction of Caspase-3/7 activity after 6 hrs treatment with 0.5 μg/ml TRAIL after knock-down of confirmed hit genes with multiple siRNAs compared to induction in cells transfected with negative control siRNA. Error bars represent 1 standard deviation (n = 3) all results are significant from negative control (P < 0.05, Students' t-test).

We next screened a library of 12,190 siRNAs, targeting the 6,095 genes of the druggable genome, excluding kinase and phosphatase genes (which were screened separately), in duplicate, for novel genes in the TRAIL-induced cell death pathway (Figure 2a and Figure 2b, complete results see Additional file 1). We undertook a careful confirmation process for genes targeted by the top 20 scoring siRNAs. Confirmation was based on two criteria. First, at least two siRNAs targeting the gene must have a significant effect in a second assay for sensitivity to TRAIL-induced apoptosis. This secondary assay took the form of a measurement of Caspase-3/7 activity levels following treatment with TRAIL. Caspases-3 and -7 are executioner Caspases and so their levels report directly on apoptosis, instead of cell death in general (as in the primary assay). Second, at least two of those siRNAs that have an effect in the secondary assay must also have an effect on target mRNA levels, and this effect must be larger than any siRNA that does not have a significant effect in the secondary assay (Figure 2c and Additional File 2, Figure S1). Thus, IGF1R is counted as a hit because two siRNAs targeting it have a significant effect on Caspase-3/7 levels, and both reduce IGF1R transcript levels, while a third siRNA has no effect on transcript levels and is thus excluded. Conversely the effect LRPAP1 siRNAs was designated as 'off-target' because although two of the three siRNAs targeting it had an effect on Caspase-3/7 induction, the siRNA which did not have a significant phenotypic effect produced a stronger knock-down at the mRNA level than either of the siRNAs that did have an effect. GPR132 is designated as 'Unconfirmed' because while the siRNA that scored highly in the screen showed a reproducible effect on Caspase-3/7 activation levels, other siRNAs targeting GPR132 did not. However, none of these other siRNAs reduced the levels of GPR132 as efficiently as the first siRNA.

In five cases (*IGF1R*, *MAX, MYC, PDE11A *and *TEGT*), we were able to confirm that the effect of siRNAs targeting these genes was due to on-target effects by these criteria, therefore implicating these genes as genuine regulators of TRAIL-induced apoptosis. In the case of *PDE11A *we were unable to detect expression in any sample (possibly due to very low wild-type expression levels), but included the gene in our hit list since we found three siRNAs targeting *PDE11A *that have a significant effect on Caspase-3/7 levels. Kinase and phosphatase genes were investigated in a separate screen, but we were unable to confirm any hits (data not shown).

*MYC *and *IGF1R *are both genes that have previously been implicated in sensitivity to TRAIL [13,14,20]. MAX is MYC's dimerization partner and is required for both the transcriptional activating and suppressing functions of MYC [28]. That these genes are amongst our hit list confirms the power of our screen for identifying genes in the TRAIL pathway. *PDE11A *encodes a dual specificity phosphodiesterase that hydrolyzes both cAMP and cGMP and is up-regulated in some carcinoma cell types [29,30]. Since cAMP is involved in the regulation of the anti-apoptotic BCL-2 via CREB and AKT, this could possibly implicate the IGF1-AKT pathway in the regulation of TRAIL induced apoptosis, in agreement with the finding that IGF1R is also involved [31].

The function of product of *TEGT *is not known, however, it has been shown to protect against several triggers of apoptosis (but not FAS), the opposite of the phenotype shown here [32]. How this factor might be involved in the regulation of TRAIL-induced apoptosis is unknown.

### Enrichment of seed sequences in high scoring siRNAs

During the confirmation of our screening results we encountered a large number of off-target effects - siRNAs which caused a phenotypic effect that was independent of their effect of the transcript level of the intended target. It is also worth noting that in several cases we identified genes where knock-down by two siRNAs induced TRAIL resistance, while a third siRNA did not, despite reducing the mRNA levels further, suggesting that the effect of both of the first two siRNAs were due to off-target effects (Additional file 2, Figure S1). This suggests that two independent siRNAs may not be sufficient for confirming hits in siRNA screens.

Lin and colleagues previously found that all three of the top siRNAs in an RNAi screen induced off-target effects, that two of these three siRNAs shared the same sequence in nucleotides 2-8 (similar to the seed sequences of miRNAs) and that the off-target effects were due to matches to these sequences [26]. To examine if a similar effect could be behind the off-target effects in our screen we examined the hexamer (nucleotides 2-7) and heptamer (nucleotides 2-8) seed sequences of the 20 highest scoring siRNAs from our screen (Table 1). Surprisingly several seed sequences appear more than once in this list. In total 3 hexamers appeared two or more times and one of these appears 4 times. Of these, 2 (CAAGGT and ACTTGA) appeared twice and three times respectively in siRNAs that caused a reproducible effect on TRAIL-induced Caspase-3/7 levels in confirmation assays. Three heptamer seed sequences appear twice in the top 20 siRNAs, one of which is found twice in siRNAs confirmed to have a reproducible effect on TRAIL-induced Caspase-3/7 levels. To test the significance of these observations we used a sampling approach (see materials and methods). Finding that two siRNAs in the top 20 share a heptamer seed sequence is significant (P < 0.045). Although observing two siRNA sharing a hexamer seed sequence is not significant at the 5% level, observing two or more such seeds is significant (P = 0.013), while finding four siRNAs sharing a seed sequence is highly significant (P < 0.0002). If only siRNAs with reproducible effects are considered, finding two heptamers (P = 0.034) or three hexamers (P = 0.001) is still significant.

**Table 1:** Seeds and Confirmation status of the top 20 siRNAs

*Transcript*	*Symbol*	*Score*	*Hexamer Seed*	*Heptamer Seed*	*Status*	*Enriched Seed*
NM_003217	*TEGT*	3.51	**CAAGGT**	**TCAAGGT**	OTE	+
NM_016368	*ISYNA1*	3.43	**TGTCCA**	**GTGTCCA**	OTE	*
NM_001240	*CCNT1*	3.28	**CAAGGT**	**TCAAGGT**	OTE	+
NM_002197	*ACO1*	3.11	**ACTTGA**	CACTTGA	Unconfirmed	+
NM_003947	*HAPIP*	2.88	**ACTTGA**	TACTTGA	OTE	+
NM_002337	*LRPAP1*	2.8	TCACAA	ATCACAA	OTE	+
NM_005799	*INADL*	2.76	**ACTTGA**	**AACTTGA**	OTE	+
NM_003554	*OR1E2*	2.72	TTGTAA	TTTGTAA	Unconfirmed	-
NM_013345	*GPR132*	2.68	AGATCA	GAGATCA	Unconfirmed	+
NM_004584	*RAD9A*	2.65	TAAGGA	CTAAGGA	OTE	-
NM_003266	*TLR4*	2.64	AAAGTT	CAAAGTT	Unconfirmed	-
NM_000674	*ADORA1*	2.63	TAATAA	CTAATAA	OTE	+
NM_002382	*MAX*	2.57	AAGAAA	TAAGAAA	Hit	-
NM_006986	*MAGED1*	2.56	**ACTTGA**	**AACTTGA**	False +ve	+
NM_000875	*IGF1R*	2.55	CACTTA	ACACTTA	Hit	-
NM_016953	*PDE11A*	2.54	GTAAGA	GGTAAGA	Hit	-
NM_014379	*KCNV1*	2.54	**TGTCCA**	**GTGTCCA**	False +ve	*
NM_080674	*C20orf86*	2.52	CGTTGT	GCGTTGT	False +ve	-
NM_002467	*MYC*	2.51	TTACAC	GTTACAC	Hit	+
XM_497793	*LOC402037*	2.51	GCATTA	GGCATTA	False +ve	-

Table shows the top 20 siRNAs from the screen, their status: Hit, False pos (did not repeat), Unconfirmed (that is this siRNA reproducibly affected caspase-3/7 activation levels, but we could not confirm the involvement of the gene targeted), or OTE (off-target effect), the hexamer and heptamer seed sequences (sequences that appear more than once are shown in bold), and whether the seed is one which either appears more than once, or is in the set of enriched seed sequences (see Table 2). * The seed sequence TGTCCA appears once in the OTE siRNA ISYNA1 and once in the false positive siRNA targeting KCNV1.

Restricting our examination of seed sequences to the top 20 siRNAs is arbitrary. The Gene Set Enrichment Analysis (GSEA) algorithm tests for the enrichment of a set of items within a larger ranked list of such items [33]. In order to gain a more general view of the enrichment of seed sequences in the high scoring siRNAs of our screen we extracted the seed sequences of every siRNA in the screen, created sets of siRNAs that shared seed sequences and applied the GSEA algorithm to identify seed sequences generally enriched in high scoring siRNAs. Using a family-wide error rate corrected P value threshold of 0.05 a total of 17 hexamer and 13 heptamer seeds were identified that were significantly enriched in high scoring siRNAs (Table 2). This finding confirms Lin *et al*'s hypothesis that screening itself enriches for siRNAs with particular seed sequences. From here on we use 'enriched seed' to refer to both those seeds identified by GSEA and those that appear multiple times in the 16 confirmed siRNAs.

**Table 2:** Seed sequences enriched in high scoring siRNAs

*Seed*	*Size*	*Normalized Enrichment Score*	*FWER p-val*
**Hexamers**			
TAATAA	70	2.691	0
ACTTGA	14	2.674	0
CCTTAA	16	2.642	0
AATTAA	44	2.567	0
ACTGGA	10	2.510	0
TAGGAA	13	2.505	0
TAAAGA	20	2.482	0.002
GAATAA	21	2.474	0.002
AAGTTA	20	2.426	0.004
TCACAA	12	2.413	0.005
AAATGA	21	2.405	0.005
AGATCA	35	2.378	0.006
TTATAA	22	2.335	0.009
AATATT	15	2.314	0.015
AGATCT	17	2.304	0.015
TGAATA	16	2.255	0.026
CTGGAA	8	2.198	0.047
**Heptamers**			
CAATTAA	18	2.911	0.000
GAGATCA	18	2.387	0.001
GAAAGAA	10	2.241	0.015
TTAATAA	17	2.227	0.015
GTATTTA	16	2.211	0.016
ATAGGAA	6	2.187	0.021
ACCTTAA	8	2.185	0.021
TAATTAA	8	2.179	0.024
TTAATTA	10	2.151	0.037
ACAATTA	10	2.148	0.039
AAGATCA	13	2.140	0.041
CTAATAA	20	2.139	0.041
CTAATTA	7	2.136	0.043

Table shows seed sequences designated enriched in high scoring siRNAs by GSEA. The number the seeds in the set, the enrichment score and the multiple testing corrected P value are shown.

**Figure 3:**
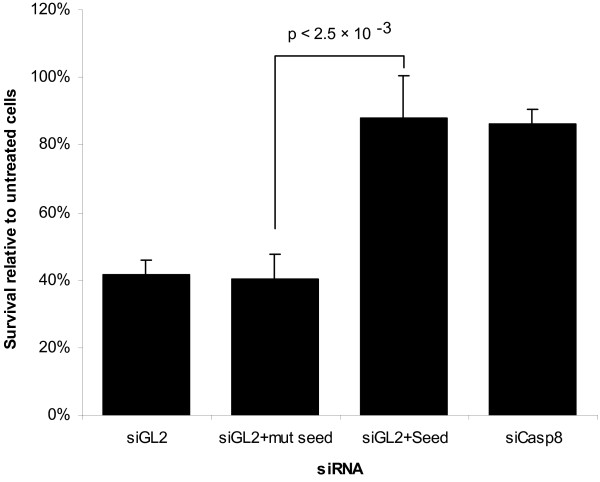
**The seed sequence ACTTGA protects cells from TRAIL-induced cell death**. HeLa cells were transfected with siRNAs targeting Caspase-8 (siCasp8), Luciferase (siGL2) or siRNAs where the siGL2 sequence had been altered to contain the seed sequence ACTTGA (siGL2+seed) or the seed sequence ATCTGA (siGL2+mutseed). After 48 hrs the sensitivity to treatment with 0.5 μg/ml TRAIL was measured. Results are shown relative to cells not treated with TRAIL. Error bars represent 1 standard deviation (n = 3 biological replicates each with 3 technical replicates). Difference between siGL2-seed and siGL2-mutseed is significant (P < 2.5 × 10-3, t-test on log-transformed data).

Since siRNAs with these seeds have a tendency to score highly, it is possible that they are scoring highly because they contain these seed-sequences rather than because the intended target is involved in TRAIL-induced apoptosis. To test if the presence of an enriched seed alone could alter the sensitivity of cells to TRAIL induced apoptosis, we transfected cells with either an siRNA targeting Luciferase (siGL2), or anti-luciferase siRNAs where the seed sequence (nucleotides 2-7) was replaced with either the sequence ACTTGA (siGL2+seed) or a version of the same sequence with two bases exchanged (ATCTGA - siGL2+mutseed). ACTTGA appears in the seed position of 3 of the 16 confirmed siRNAs, and was the second most enriched seed sequenced identified by GSEA. Cells transfected with the siRNA containing the mutated seed have a very similar sensitivity to TRAIL-induced cell death to that of cells transfected with the unaltered anti-Luciferase siRNA (Figure 3). However, cells transfected with the siRNA containing ACTTGA had a sensitivity close to that of cells transfected with an anti-Caspase-8 siRNA. Thus the presence of the seed sequence ACTTGA alone in an otherwise non-effective siRNA can have a strong effect on sensitivity to TRAIL-induced cell death.

In total 9 of the 16 siRNAs with a confirmed effect on Caspase-3/7 actiavation contain a seed from one of these two sets of sequences, including six of the top 7 siRNAs. This includes two siRNAs targeting genes that had been selected as confirmed hits (siRNAs targeting *TEGT *and *MYC*). In each of these cases we had found a second siRNA targeting the gene that also had a significant effect on TRAIL-induced apoptosis. However, in the case of *TEGT *that other siRNA (which was the second siRNA used in the screen) also contained one of the enriched seed sequences. Given the amount of evidence for the involvement of MYC in the TRAIL pathway from the literature, it is unlikely that siRNAs targeted against *MYC *exert their effect solely though off-target effects [13,34-38]. Testing further siRNAs targeting TEGT we were unable to confirm the involvement of TEGT in TRAIL-induced apoptosis (Additional file 2, Figure S2), thus changing the status of TEGT siRNAs from "hit" to "off-target".

Of the 20 highest scoring siRNAs, 16 had a statistically significant effect on TRAIL-induced cell death. The effects of eight siRNAs could be shown to be due to off-target effects, of which 6 (75%) have one of the enriched seed sequences. In total the effects of the 12 of the 16 (75%) siRNAs which reproducibly reduced the sensitivity of cells to TRAIL-induced cell death could be attributed to either the targeting of a hit gene or the seed sequence of the siRNA. We cannot say that all the genes targeted by an siRNA containing a seed are definitely not involved in TRAIL-induced apoptosis, just that at least part of their effect is likely to be due to off-target effects. Even so the majority of siRNAs in the top 20 appear to owe at least part of their activity to off-target effects. Such findings suggest that screening enriches for siRNAs with off-target effects and that a high scoring siRNA in this assay is more likely to exert its effect through an off-target effect than by affecting the intended target. Thus off-target effects are probably more widespread than is generally recognised.

The usual method for distinguishing true hits from RNAi screens from off-targets is to require that two independent siRNAs targeting the same gene have the same phenotype [39]. However, one clear implication of this study is that this is insufficient, particularly when both siRNAs were used in the screen (as for *TEGT *in this study). We recommend that hits should be confirmed using at least two, and ideally three or more siRNAs not used in the screen, and whose seeds have been checked.

Finally, we note that not only can seed sequences lead to a substantial number of false positives in RNAi screens, but can also mask true hits and thus increase false negative rates. In the TRAIL-killing screen described here, we tested for the enrichment of genes previously associated with the TRAIL pathway. We identified 27 genes that had previously been associated with the TRAIL induced apoptosis pathway and knockdown of which might be expected to decrease sensitivity to TRAIL-induced cell death. siRNAs against 12 of these were present in our library (BID, MYC, TNFRSF10A, TNFSRF10B, CASP8, DVL2, FBXO11, BAX, CASP3, TCF4, FADD and VPS16). We applied GSEA's pre-ranked analysis to test for enrichment of this gene set in the results from our screen. We found no significant trend, suggesting that we had failed to pick up most of the previous known genes involved in TRAIL killing (normalized enrichment score (NES) = 1.64, P = 0.097). To determine if this was due to a real effect being hidden by the off-target effects, we removed any siRNA containing an enriched seed sequence from our data and reanalysed the screening data. The ranking of siRNAs under this regime is available in Additional File 3. Under this new analysis regime, there was a significant enrichment of genes previously identified as being involved in TRAIL-induced apoptosis when GSEA was used to test for enrichment of gene set described above. (NES = 1.64, P = 0.02). Thus, dividing screen hits according to on and off-target effects not only reduces noise in the screens but also can result in significant gains in sensitivity as well.

### Identification of miRNAs affecting sensitivity to TRAIL-induced apoptosis

Transfecting siRNAs to knock-down target genes is equivalent to over-expressing miRNAs of the same sequence as the siRNA. If many of the siRNAs that score well in our screen are exerting their effect through miRNA-like effects determined by a set of common seed sequences we reasoned that we might be able identify miRNAs with the same seed sequences and this might be an indication that these miRNAs are involved in regulating TRAIL-induced apoptosis. We searched a set of seed sequences found in human miRNAs downloaded from the miRBase database (version 10.0). We identified three miRNAs whose seed sequences were the same as those identified above (Table 3).

**Table 3:** Enriched seeds that are present in human miRNAs

*Seed*	*miRNA*
ACTTGA	has-miR-26a
ACTGGA	has-miR-145
TAGGAA	has-miR-384

One of these miRNAs (miR-145) has previously been implicated in the regulation of TRAIL induced apoptosis [21]. miRNA-26a has also been implicated in regulation of Caspase-3 levels [21]. MiR-26a is highly expressed in chronic lymphocytic leukemia cell lines which are often resistant to TRAIL-induced apoptosis, while miR-145 is down-regulated in colorectal and prostate carcinomas, which are generally sensitive to TRAIL-induced apoptosis [40-43]. Interestingly it has recently been shown that miR-26a is repressed by MYC [44]. Very little is known about miR-384.

**Figure 4:**
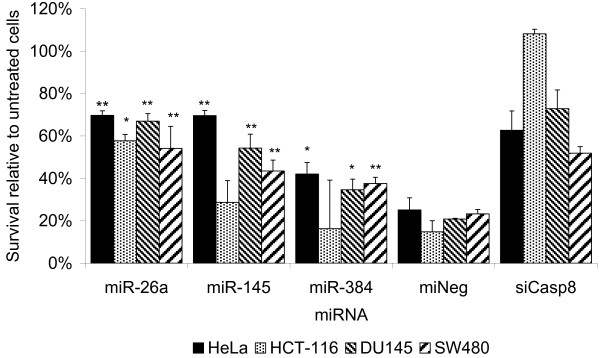
**miRNAs containing enriched seeds protect a several cell types from TRAIL induced cell death**. Cells were transfected with miRNA mimics with same sequence as naturally occurring miRNAs that contain seed sequences enriched in high-scoring siRNAs, an miRNA mimic with a sequence occurring in no human miRNA, or an siRNA targeting Caspase-8 as a positive control. After 48 hrs the sensitivity to treatment with 0.5 μg/ml TRAIL was measured. Error bars represent 1 standard deviation (n = 3 bioligical replicates each with 3 technical replicates, * represents results significantly different from the negative control (P < 0.05, students' T-test on log-transformed data), ** are significantly different after Bonferroni correction.

To confirm of the ability of these miRNA to affect sensitivity to TRAIL, we transfected HeLa cells with mimics of these miRNAs and tested the sensitivity of these cells to TRAIL-induced cell death (Figure 4). Mimics of all three miRNAs had a significant effect on the sensitivity of cells to TRAIL-induced cell death compared to cells transfected with a mimic of a *Caenorhabditis elegans *miRNA used as a negative control. In two of the three cases (miR-26a and miR-145) this difference was still significant after correction for multiple testing. To test if the involvement of these miRNAs was specific to HeLa cells or extends to other cell types, we transfected DU145 (prostate carcinoma), HCT-116 and SW480 (both colorectal carcinoma) cells with the same miRNA mimics. The effects of the miRNA mimics were similar in SW480 and DU145 cells to HeLa cells, while in HCT-116 the effects were significant only in the case of the mimic of miR-126a (Figure 4). This demonstrates that these miRNAs may have an effect in regulating TRAIL-induced cell death that extends beyond HeLa cells. To the best of our knowledge this is the first time that the involvement of miRNAs in a process has been suggested from studying RNAi off-target effects.

## Conclusions

We have executed a screen for regulators of the TRAIL induced apoptosis pathway and identified a novel gene involved in this pathway: *PDE11A*. We also confirmed the involvement of several genes previously identified in connection with the TRAIL-induced apoptosis pathway. The small size of the overlap with previous screens suggests that these screens are not saturating and that other genes involved in the pathway remain to be identified.

Most previous reports of siRNAs functioning through miRNA-like targeting mechanisms have reported isolated examples of such effects. We identified a set of seed sequences that are over-represented in siRNAs identified as potential hits from the screen and a further set of seed sequences that is generally enriched in high-scoring siRNAs. We showed that one of these seed sequences is sufficient to render an otherwise inactive siRNA active in our assay. This suggests that siRNAs containing one of these seed sequences exert their effects due, at least in part, to the presence of one of these seed sequences. The use of a statistical enrichment approach to identify seed sequences which are more common in highly scoring siRNAs provides a more complete picture of the influence of seed sequence driven off-target effects on screen results.

Where as previously off-target events have been regarded as unfortunate anomalies (which can in some circumstances be driven by miRNA-like targeting through seed sequences), our findings provides support for the hypothesis of Lin *et al*, that RNAi screens enrich for siRNAs with off target effects and suggests that any siRNA scoring highly in a screen is very likely to be exerting at least part of its effect through off-target effect. This suggests that a gene targeted by two highly scoring siRNAs in a screen may still be an off-target hit as was the case here with *TEGT*. We recommend that in future all screening results be regarded with suspicion until they are confirmed by at least two siRNAs (preferably more) not used in the original screen, and do not contain seed sequences found in high scoring siRNAs from the screen.

Removing siRNAs containing enriched seed sequences allowed us to show that siRNAs targeting genes known to be involved in the TRAIL induced apoptosis pathway were enriched in high-scoring siRNAs. This suggests that identifying enriched seed sequences and removing siRNAs containing them from analysis in the future will aid with such investigations.

One obvious approach to combating such off-target effects would be to exclude siRNA with common seed sequences at the design stage (as is the case for at least one commercial siRNA design algorithm). While this may exclude siRNAs with the largest number of off-target effects (although it clearly won't prevent all seed-driven off-target effects), seeds from endogenous miRNAs are not necessarily the ones that are most common in 3' UTRs. While siRNAs containing the seeds of endogenous miRNAs may have fewer targets, their targets are likely to have some degree of functional relation, and are therefore more likely to have a strong functional effect on the cell. Excluding siRNAs containing the top 10% of 6 mer seed sequence by frequency would have removed 6 of the 17 seed sequences we found to be enriched, but none of the seed sequences we identified that are found in endogenous miRNAs (data not shown). Excluding so many seed sequences also greatly reduces the design space for siRNAs and will result is more siRNAs sharing the same seed sequences (and, if off-target effects are driven by seed sequences, the same off-target effects).

Finally we used the enriched seed sequences to identify miRNAs that may be involved in TRAIL-induced apoptosis, identifying miRNAs miR-26a, miR-145 and miR-384 as affecting TRAIL-induced cell death in a range of cell types. Further work will be needed to examine if these miRNAs have an endogenous role in regulating the TRAIL-induced apoptosis pathway. This analysis allows additional information to be gathered from the results of RNAi screening and turns potentially confounding off-target effects into a new source of information for the process being studied.

miRNAs present an attractive candidate for controllers of sensitivity to TRAIL-induced apoptosis as they can control many genes simultaneously, allowing those changes necessary to transform cells to be linked to changes sensitizing them to TRAIL-induced apoptosis.

## Methods

### Cells and cell culture

HeLa cells were obtained from ATCC (#CCL-2) and were maintained in Modified Eagle's medium (Sigma-Aldrich) supplemented with 10% Fetal Bovine Serum, 2 mM L-Glutamine, 1× Non essential amino acids, penicillin and streptomycin. SW480, HCT116 and DU145 cells were a kind gift from Prof. Mike Stratton. DU145 cells were maintained in the same media as HeLa cells. SW480 cells were maintained in Leibovitz's L-15 Medium (Sigma-Aldrich) supplemented with 10% Fetal Bovine Serum, 2 mM L-Glutamine, penicillin and streptomycin. HCT116 cells were maintained in McCoy's 5a (Sigma-Aldrich) supplemented with 10% Fetal Bovine Serum, 2 mM L-Glutamine, penicillin and streptomycin.

### siRNAs, miRNA mimics and transfection

Individual siRNAs were obtained from either Invitrogen, Qiagen, Ambion or Dharmacon. Sequences and suppliers of siRNAs is listed in additional file 4. miRNA mimics were obtained from Dharmacon. Library siRNAs were from the Qiagen Druggable Genome Library v2.

Cells were transfected with 2.5 pmol siRNA in 96-well plates or 12.5 pmol siRNA in 24-well plates using Lipofectamine 2000 (Invitrogen) according to manufacturer's instructions for each cell type.

### Assay for sensitivity to TRAIL-induced apoptosis

For HeLa cells, 3,000 cells were seeded into the well of a 96 well optilux plate (BD Bioscience), in antibiotic free media. 24 hours later cells were transfected with siRNA. After a further 48 hours the number of cells present was measured by incubating for 3 hours with 10% alamarBlue reagent (AbD Serotec, Oxford, UK) before reading flourescence (excitation: 544 nm, emission: 590 nm) using a fluorescent plate reader (Molecular Devices, Sunnyvale, CA). Cells were treated with 0.5 μg/ml recombinant TRAIL (Calbiochem) in serum-free media for 20 hours and number of cells present was reanalysed as above.

### siRNA screen

12,190 siRNAs from the Qiagen druggable genome library v2 were arrayed into 96-wells plates along with two duplicates of the siRNAs targeting Caspase-8, *BID *and *DIABLO *as positive controls, two duplicates of an siRNA targeting siKIF11 as a transfection control, and four wells containing three different negative controls (siNeg1, siNeg2 and siGFP). The siRNAs were transfected into HeLa cells and the effect on TRAIL induced measured as described above. The screen was performed in duplicate. Plate were quality controlled by measuring the dynamic range between positive control transfected wells and negative control transfected wells, plates where the positive control was not twice the negative control were repeated.

Data analysis was performed using the cellHTS package in R/Bioconductor [45]. Briefly, the bottom 20% wells for cell survival before TRAIL treatment were removed from analysis. Survival was calculated by dividing post treatment fluorescence by pre-treatment flourescence and the resulting ratio log-transformed and normalized to plate median. Each well was converted to a z score using the median and median absolute deviation for each repeat:

The minimum of the two scores for each siRNA was selected to represent that siRNA. See cellHTS report in supplementary information for analysis script.

### Confirmation of screen hits

For each gene targeted by an siRNA in the top 20 scoring siRNAs, both siRNAs in the library were retested in triplicate. Where only one siRNA targeting a gene had a significant effect a further two siRNAs were obtained. siRNAs were tested for their effect on Caspase-3/7 activity. 3,000 cells were seeded in optilux plates and transfected with siRNA as described above. 48 hours after transfection cells were incubated with 0.5 μg/ml recombinant TRAIL in serum free media for 6 hours and the level of Caspase-3/7 measured using the Caspase-glo 3/7 kit (Promega) according to manufacturer's instructions.

To measure the effect of siRNAs on mRNA levels, 15,000 cells were transfected with 12.5 pmol siRNA using 0.6 μl Lipofectamine 2000 and after 48 hours RNA prepared using the SV Total RNA isolated kit (Promega), and cDNA prepared using Superscript II (Invitrogen). SYBR green qPCR was performed using qPCR MasterMix (Eurogentec) with primers designed to amplify the transcript of interest or ACTB or GAPDH. All primers were tested to measure amplification efficiency. See additional file 5 for primer sequences. Relative quantities were calculated using the qBase software [46].

### Identification of enriched seed sequences

To test the significance of observing the same seed sequence in multiple siRNA from the top 20 siRNAs, the seed sequence of all siRNAs in the library were extracted. Samples of twenty seed sequences were selected at random, and the number of times seed sequences appeared counted. This was repeated 5,000 times for both hexamer and heptamer seed sequences and the number of times any seed sequence appeared more than one was counted.

To use GSEA to detect enriched seed sequences, "gene-sets" were built containing the each of the siRNAs containing at particular seed sequence. The GSEA desktop application's pre-ranked analysis option was used to test if these sets were enriched in high-scoring siRNAs. This was repeated for both hexamer and heptamer seed sequences.

## Abbreviations

GSEA: Gene Set Enrichment Analysis; miRNA: microRNA; RNAi: RNA interference; siRNA: small interfering RNA; TRAIL: TNF related apoptosis inducing ligand.

## Authors' contributions

IMS was involved in the conception, design and interpretation of the work, carried out all experimental work, screening data analysis, identification of seed sequences and wrote the manuscript. AJE was involved in the interpretation of the data, the analysis of the enriched seed sequences and editing the manuscript. AGF and ID were involved in the conception, design and interpretation of the work and editing the manuscript. The final manuscript has been reviewed and approved by all authors.

## Supplementary Material

Additional file 1 **Complete screen results (screen_results.zip)**. Complete results from the siRNA, presented as a mini-website as produced by the cellHTS software Click here for file

Additional file 2 **Supplementary figures (supplementary_figures.pdf)**. Figures S1 and S2 containing detailed results from confirmation experiments for all 16 genes targeted by siRNAs from the screen which reproducibly reduce sensitivity to TRAIL-induced cell death. Click here for file

Additional file 3 **Ranking of siRNAs after removal of siRNAs with enriched seed sequences (no_enriched_seeds.csv)**. Ranking of siRNAs used in the screen according to their score, after any siRNA containing an enriched seed had been removed and data reanalysed. Click here for file

Additional file 4 **Sequences of siRNAs used (siRNAs.csv)**. List of sequences, suppliers and catalogue numbers for non-library siRNAs. Click here for file

Additional file 5 **Sequences of qRT-PCR primers (primers.csv)**. List of primer sequences used along with product lengths and amplification efficiency for each pair. Click here for file
