# Supplementary material for: Systematic analysis of off-target effects in an RNAi screen reveals microRNAs affecting sensitivity to TRAIL-induced apoptosis
Source: BMC Genomics. 2010 Mar 15;11:175. doi: 10.1186/1471-2164-11-175 (PMC2996961; doi:10.1186/1471-2164-11-175)
Supplement: Additional file 1 — Complete screen results (screen_results.zip). Complete results from the siRNA, presented as a mini-website as produced by the cellHTS software [file 1471-2164-11-175-S1.ZIP › 153/index.html]

Quality report for Plate 153 (normalized)


# Quality report for Plate 153 (normalized)


| Channel 1 | | |
| --- | --- | --- |
| metric | value | comment |
| Dynamic range 'siBID' (replicate 1) | 1.09 |  |
| Dynamic range 'siBID' (replicate 2) | 0.35 |  |
| Dynamic range 'siBID' | 0.72 |  |
| Dynamic range 'siCasp8' (replicate 1) | 1.45 |  |
| Dynamic range 'siCasp8' (replicate 2) | 1.66 |  |
| Dynamic range 'siCasp8' | 1.55 |  |
| Dynamic range 'siSMAC' (replicate 1) | 0.61 |  |
| Dynamic range 'siSMAC' (replicate 2) | 1.32 |  |
| Dynamic range 'siSMAC' | 0.36 |  |
| Spearman rank correlation | 0.48 |  |

  
  


|  | Channel 1 |
| --- | --- |
|  | SCATTERPLOT BETWEEN REPLICATES |
|  | flagged: 27  *Color legend:*   pos: 6, neg: 4, sample: 57, other: 2, empty: 0 |
|  | HISTOGRAM(S) |
| Replicate 1 |  |
| Replicate 2 |  |
|  | PLATE PLOT(S) |
| Standard deviation across replicates |  |
| Replicate 1 |  |
| Replicate 2 |  |

  

---

Sat Nov 10 17:37:08 2007
