# Supplementary material for: Systematic analysis of off-target effects in an RNAi screen reveals microRNAs affecting sensitivity to TRAIL-induced apoptosis
Source: BMC Genomics. 2010 Mar 15;11:175. doi: 10.1186/1471-2164-11-175 (PMC2996961; doi:10.1186/1471-2164-11-175)
Supplement: Additional file 2 — Supplementary figures (supplementary_figures.pdf). Figures S1 and S2 containing detailed results from confirmation experiments for all 16 genes targeted by siRNAs from the screen which reproducibly reduce sensitivity to TRAIL-induced cell death. [file 1471-2164-11-175-S2.PDF]

a)

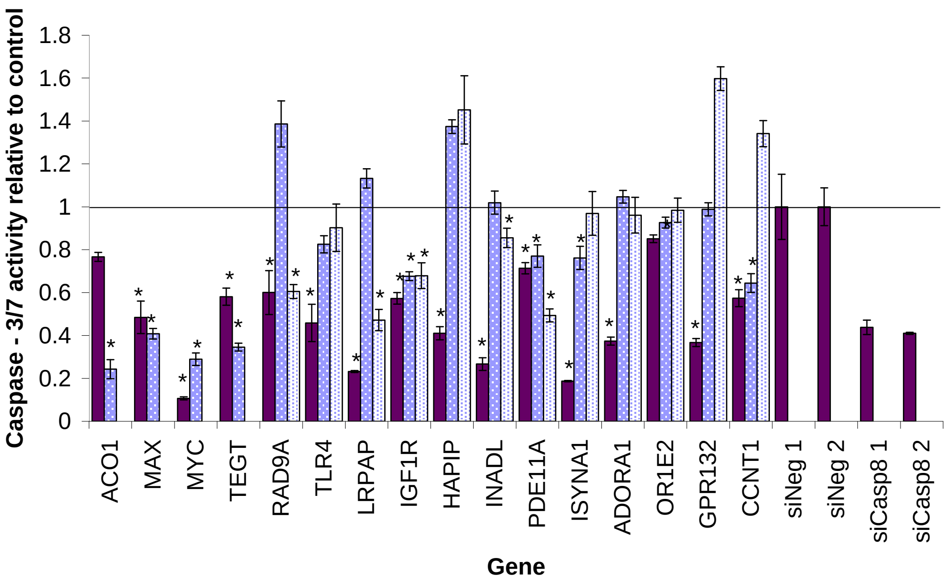

c)

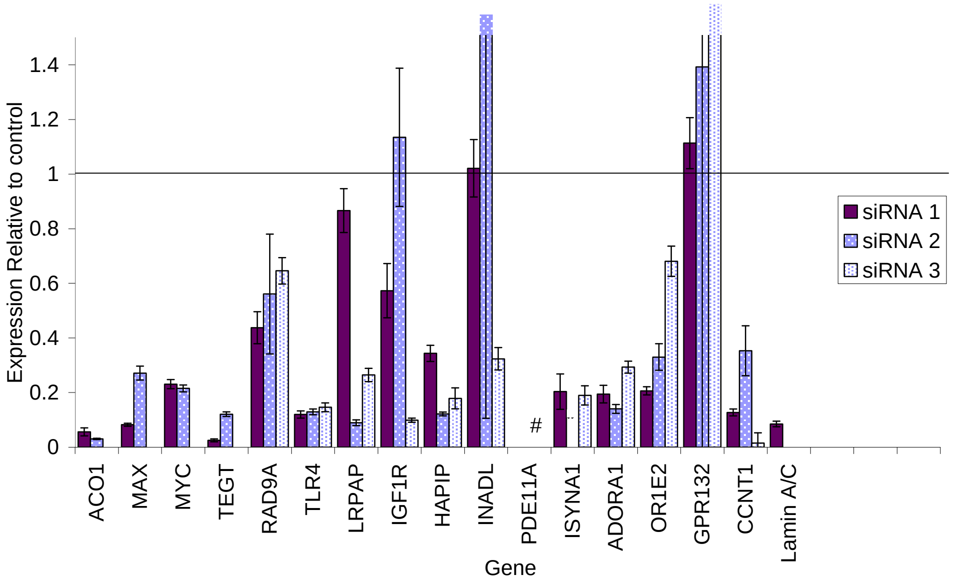

Figure S1 – Confirmation of hits from screen. Hit genes from the screen where the effect of at least one siRNA could be replicated were selected for confirmation. Where necessary additional siRNAs targeting the genes were obtained. a) Induction of Caspase – 3/7 activity upon treatment with 0,5µg/ml TRAIL after knock-down of potential hits compared to induction in negative control. Different coloured bars represent different siRNAs targeting the same genes. Results are shown relative to negative control. Error bars represent one standard deviation (n=3). * represents effects significantly different from negative control (P<-0.05, students' t-test). siNeg pl1/siNeg pl2 and siCasp8 pl1/siCasp pl2 represent positive and negative controls on the two separate plates in the experiment. b) mRNA levels of potential hit genes after knock-down. Bars represent same siRNAs as in a). Expression is shown relative to levels cells transfected with negative control siRNA. siRNA used to knock-down Lamin A/C used as a positive control. Error bars represent 1 standard deviation (n=3 PCR reactions). # could not amplify. Solid line in each panel represent negative control level.

a)

b)

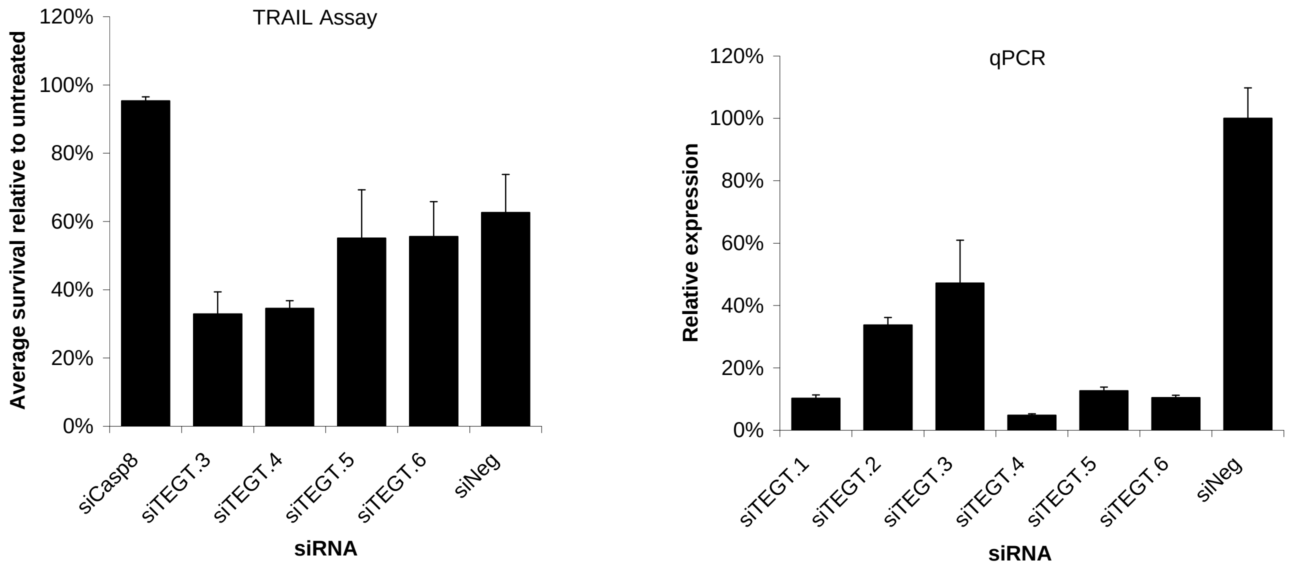

Figure S2 - Investigation of extra siRNAs targeting *TEGT*. a)Effect of knockdown of *TEGT* by 4 novel siRNAs on sensitivity to treatment with 0.5µg/ml TRAI. Results are shown relative cells untreated with TRAIL, Error bars represent 1 standard deviation (n = 2 biological replicates each with three technical replicates) b) Effect of same siRNAs (plus siTEGT.1 and siTEGT.2) on *TEGT* mRNA levels. Error bars represent 1 standard deviation (n=3 PCRs).
