# Supplementary material for: Systematic analysis of off-target effects in an RNAi screen reveals microRNAs affecting sensitivity to TRAIL-induced apoptosis
Source: BMC Genomics. 2010 Mar 15;11:175. doi: 10.1186/1471-2164-11-175 (PMC2996961; doi:10.1186/1471-2164-11-175)
Supplement: Additional file 1 — Complete screen results (screen_results.zip). Complete results from the siRNA, presented as a mini-website as produced by the cellHTS software [file 1471-2164-11-175-S1.ZIP › Controls_2_1.pdf]

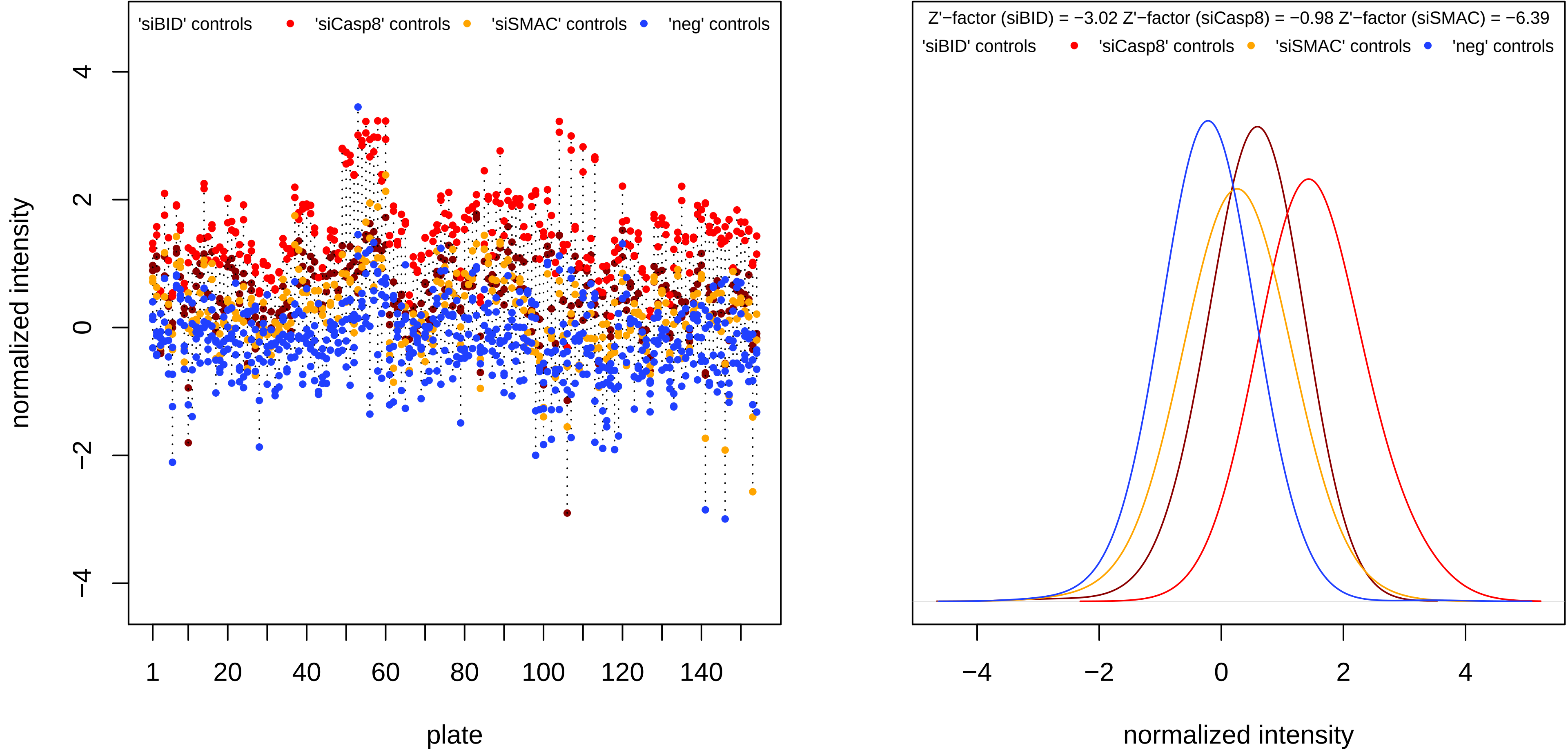
'siBID' controls
'siCasp8' controls
'siSMAC' controls
'neg' controls
normalized intensity
plate
-4
-2
0
2
4
1
20
40
60
80
100
120
140
Z'−factor (siBID) = −3.02 Z'−factor (siCasp8) = −0.98 Z'−factor (siSMAC) = −6.39
normalized intensity
