# Supplementary material for: Systematic analysis of off-target effects in an RNAi screen reveals microRNAs affecting sensitivity to TRAIL-induced apoptosis
Source: BMC Genomics. 2010 Mar 15;11:175. doi: 10.1186/1471-2164-11-175 (PMC2996961; doi:10.1186/1471-2164-11-175)
Supplement: Additional file 1 — Complete screen results (screen_results.zip). Complete results from the siRNA, presented as a mini-website as produced by the cellHTS software [file 1471-2164-11-175-S1.ZIP › index.html]

Experiment report for TRAIL\_Druggable\_Genome\_Screen


# Experiment report for TRAIL\_Druggable\_Genome\_Screen


| Filename | status | Plate | Replicate | Channel | Batch | Replicate dynamic range (siBID) | Replicate dynamic range (siCasp8) | Replicate dynamic range (siSMAC) | Average dynamic range (siBID) | Average dynamic range (siCasp8) | Average dynamic range (siSMAC) | Spearman rank correlation |
| --- | --- | --- | --- | --- | --- | --- | --- | --- | --- | --- | --- | --- |
| 01a1.pda.txt.before | OK | 1 | 1 | 1 | 1 | 1.25 | 2.03 | 1.44 | 1.04 | 1.6 | 1.05 | 0.62 |
| 01a1.pda.txt.after | OK | 1 | 1 | 2 | 1 |  |  |  |  |  |  |  |
| 01a2.pda.txt.before | OK | 1 | 2 | 1 | 1 | 0.83 | 1.18 | 0.65 | 1.04 | 1.6 | 1.05 | 0.62 |
| 01a2.pda.txt.after | OK | 1 | 2 | 2 | 1 |  |  |  |  |  |  |  |
| 01b1.pda.txt.before | OK | 2 | 1 | 1 | 1 | 1.06 | 1.83 | 0.32 | 1.17 | 1.8 | 0.57 | 0.8 |
| 01b1.pda.txt.after | OK | 2 | 1 | 2 | 1 |  |  |  |  |  |  |  |
| 01b2.pda.txt.before | OK | 2 | 2 | 1 | 1 | 1.28 | 1.78 | 0.82 | 1.17 | 1.8 | 0.57 | 0.8 |
| 01b2.pda.txt.after | OK | 2 | 2 | 2 | 1 |  |  |  |  |  |  |  |
| 02a1.pda.txt.before | OK | 3 | 1 | 1 | 1 | 0.6 | 1.7 | 0.36 | 0.14 | 1.16 | 0.26 | 0.2 |
| 02a1.pda.txt.after | OK | 3 | 1 | 2 | 1 |  |  |  |  |  |  |  |
| 02a3.pda.txt.before | OK | 3 | 2 | 1 | 1 | 0.31 | 0.62 | 0.16 | 0.14 | 1.16 | 0.26 | 0.2 |
| 02a3.pda.txt.after | OK | 3 | 2 | 2 | 1 |  |  |  |  |  |  |  |
| 02b1.pda.txt.before | OK | 4 | 1 | 1 | 1 | 0.15 | 0.73 | 0.66 | 0.5 | 1.28 | 0.03 | 0.59 |
| 02b1.pda.txt.after | OK | 4 | 1 | 2 | 1 |  |  |  |  |  |  |  |
| 02b2.pda.txt.before | OK | 4 | 2 | 1 | 1 | 0.85 | 1.82 | 0.72 | 0.5 | 1.28 | 0.03 | 0.59 |
| 02b2.pda.txt.after | OK | 4 | 2 | 2 | 1 |  |  |  |  |  |  |  |
| 03a1.pda.txt.before | OK | 5 | 1 | 1 | 1 | 0.31 | 1.27 | 0.69 | 0.54 | 1.43 | 0.07 | 0.51 |
| 03a1.pda.txt.after | OK | 5 | 1 | 2 | 1 |  |  |  |  |  |  |  |
| 03a2.pda.txt.before | OK | 5 | 2 | 1 | 1 | 0.76 | 1.59 | 0.55 | 0.54 | 1.43 | 0.07 | 0.51 |
| 03a2.pda.txt.after | OK | 5 | 2 | 2 | 1 |  |  |  |  |  |  |  |
| 03b1.pda.txt.before | OK | 6 | 1 | 1 | 1 | 0.22 | 0.71 | 1.06 | 0.45 | 1.16 | 0.09 | 0.44 |
| 03b1.pda.txt.after | OK | 6 | 1 | 2 | 1 |  |  |  |  |  |  |  |
| 03b3.pda.txt.before | OK | 6 | 2 | 1 | 1 | 1.13 | 1.61 | 0.87 | 0.45 | 1.16 | 0.09 | 0.44 |
| 03b3.pda.txt.after | OK | 6 | 2 | 2 | 1 |  |  |  |  |  |  |  |
| 04a1.pda.txt.before | OK | 7 | 1 | 1 | 1 | 0.4 | 0.94 | 0.45 | 0.44 | 1.06 | 0.01 | 0.6 |
| 04a1.pda.txt.after | OK | 7 | 1 | 2 | 1 |  |  |  |  |  |  |  |
| 04a2.pda.txt.before | OK | 7 | 2 | 1 | 1 | 0.47 | 1.18 | 0.48 | 0.44 | 1.06 | 0.01 | 0.6 |
| 04a2.pda.txt.after | OK | 7 | 2 | 2 | 1 |  |  |  |  |  |  |  |
| 04b1.pda.txt.before | OK | 8 | 1 | 1 | 1 | 0.26 | 0.76 | 0.75 | 0.1 | 0.96 | 0.08 | 0.81 |
| 04b1.pda.txt.after | OK | 8 | 1 | 2 | 1 |  |  |  |  |  |  |  |
| 04b2.pda.txt.before | OK | 8 | 2 | 1 | 1 | 0.46 | 1.15 | 0.58 | 0.1 | 0.96 | 0.08 | 0.81 |
| 04b2.pda.txt.after | OK | 8 | 2 | 2 | 1 |  |  |  |  |  |  |  |
| 05a1.pda.txt.before | OK | 9 | 1 | 1 | 1 | 0.17 | 0.94 | 0.53 | 0.37 | 0.95 | 0.33 | 0.67 |
| 05a1.pda.txt.after | OK | 9 | 1 | 2 | 1 |  |  |  |  |  |  |  |
| 05a3.pda.txt.before | OK | 9 | 2 | 1 | 1 | 0.58 | 0.97 | 0.12 | 0.37 | 0.95 | 0.33 | 0.67 |
| 05a3.pda.txt.after | OK | 9 | 2 | 2 | 1 |  |  |  |  |  |  |  |
| 05b1.pda.txt.before | OK | 10 | 1 | 1 | 1 | 0.12 | 0.07 | 1.21 | 0.57 | 0.65 | 0.42 | 0.17 |
| 05b1.pda.txt.after | OK | 10 | 1 | 2 | 1 |  |  |  |  |  |  |  |
| 05b3.pda.txt.before | OK | 10 | 2 | 1 | 1 | 1.26 | 1.24 | 0.37 | 0.57 | 0.65 | 0.42 | 0.17 |
| 05b3.pda.txt.after | OK | 10 | 2 | 2 | 1 |  |  |  |  |  |  |  |
| 06a1.pda.txt.before | OK | 11 | 1 | 1 | 1 | 0.38 | 0.8 | 1.11 | 0.55 | 1.2 | 0.35 | 0.15 |
| 06a1.pda.txt.after | OK | 11 | 1 | 2 | 1 |  |  |  |  |  |  |  |
| 06a3.pda.txt.before | OK | 11 | 2 | 1 | 1 | 0.73 | 1.61 | 0.42 | 0.55 | 1.2 | 0.35 | 0.15 |
| 06a3.pda.txt.after | OK | 11 | 2 | 2 | 1 |  |  |  |  |  |  |  |
| 06b1.pda.txt.before | OK | 12 | 1 | 1 | 1 | 0.45 | 0.76 | 0.48 | 0.69 | 1.03 | 0.1 | 0.7 |
| 06b1.pda.txt.after | OK | 12 | 1 | 2 | 1 |  |  |  |  |  |  |  |
| 06b2.pda.txt.before | OK | 12 | 2 | 1 | 1 | 0.94 | 1.31 | 0.28 | 0.69 | 1.03 | 0.1 | 0.7 |
| 06b2.pda.txt.after | OK | 12 | 2 | 2 | 1 |  |  |  |  |  |  |  |
| 07a1.pda.txt.before | OK | 13 | 1 | 1 | 1 | 0.46 | 0.84 | 0.12 | 0.77 | 1.2 | 0.34 | 0.81 |
| 07a1.pda.txt.after | OK | 13 | 1 | 2 | 1 |  |  |  |  |  |  |  |
| 07a2.pda.txt.before | OK | 13 | 2 | 1 | 1 | 1.09 | 1.57 | 0.55 | 0.77 | 1.2 | 0.34 | 0.81 |
| 07a2.pda.txt.after | OK | 13 | 2 | 2 | 1 |  |  |  |  |  |  |  |
| 07b1.pda.txt.before | OK | 14 | 1 | 1 | 1 | 0.45 | 0.99 | 0.6 | 0.72 | 1.46 | 0.67 | 0.76 |
| 07b1.pda.txt.after | OK | 14 | 1 | 2 | 1 |  |  |  |  |  |  |  |
| 07b2.pda.txt.before | OK | 14 | 2 | 1 | 1 | 0.99 | 1.93 | 0.73 | 0.72 | 1.46 | 0.67 | 0.76 |
| 07b2.pda.txt.after | OK | 14 | 2 | 2 | 1 |  |  |  |  |  |  |  |
| 08a1.pda.txt.before | OK | 15 | 1 | 1 | 1 | 0.61 | 1.14 | 0.29 | 0.69 | 1.31 | 0.37 | 0.67 |
| 08a1.pda.txt.after | OK | 15 | 1 | 2 | 1 |  |  |  |  |  |  |  |
| 08a2.pda.txt.before | OK | 15 | 2 | 1 | 1 | 0.78 | 1.49 | 0.44 | 0.69 | 1.31 | 0.37 | 0.67 |
| 08a2.pda.txt.after | OK | 15 | 2 | 2 | 1 |  |  |  |  |  |  |  |
| 08b1.pda.txt.before | OK | 16 | 1 | 1 | 1 | 0.34 | 0.91 | 0.4 | 0.64 | 1.16 | 0.56 | 0.72 |
| 08b1.pda.txt.after | OK | 16 | 1 | 2 | 1 |  |  |  |  |  |  |  |
| 08b2.pda.txt.before | OK | 16 | 2 | 1 | 1 | 0.93 | 1.42 | 0.72 | 0.64 | 1.16 | 0.56 | 0.72 |
| 08b2.pda.txt.after | OK | 16 | 2 | 2 | 1 |  |  |  |  |  |  |  |
| 09a1.pda.txt.before | OK | 17 | 1 | 1 | 1 | 0.18 | 0.65 | 0.14 | 0.36 | 1.13 | 0.2 | 0.76 |
| 09a1.pda.txt.after | OK | 17 | 1 | 2 | 1 |  |  |  |  |  |  |  |
| 09a2.pda.txt.before | OK | 17 | 2 | 1 | 1 | 0.55 | 1.62 | 0.27 | 0.36 | 1.13 | 0.2 | 0.76 |
| 09a2.pda.txt.after | OK | 17 | 2 | 2 | 1 |  |  |  |  |  |  |  |
| 09b1.pda.txt.before | OK | 18 | 1 | 1 | 1 | 1.19 | 1.32 | 0.58 | 1.08 | 1.49 | 0.42 | 0.79 |
| 09b1.pda.txt.after | OK | 18 | 1 | 2 | 1 |  |  |  |  |  |  |  |
| 09b2.pda.txt.before | OK | 18 | 2 | 1 | 1 | 0.96 | 1.65 | 0.27 | 1.08 | 1.49 | 0.42 | 0.79 |
| 09b2.pda.txt.after | OK | 18 | 2 | 2 | 1 |  |  |  |  |  |  |  |
| 10a1.pda.txt.before | OK | 19 | 1 | 1 | 1 | 0.28 | 0.58 | 0.5 | 0.42 | 0.98 | 0.09 | 0.7 |
| 10a1.pda.txt.after | OK | 19 | 1 | 2 | 1 |  |  |  |  |  |  |  |
| 10a2.pda.txt.before | OK | 19 | 2 | 1 | 1 | 0.57 | 1.39 | 0.32 | 0.42 | 0.98 | 0.09 | 0.7 |
| 10a2.pda.txt.after | OK | 19 | 2 | 2 | 1 |  |  |  |  |  |  |  |
| 10b1.pda.txt.before | OK | 20 | 1 | 1 | 1 | 0.79 | 1.32 | 0.3 | 0.93 | 1.6 | 0.09 | 0.82 |
| 10b1.pda.txt.after | OK | 20 | 1 | 2 | 1 |  |  |  |  |  |  |  |
| 10b2.pda.txt.before | OK | 20 | 2 | 1 | 1 | 1.06 | 1.89 | 0.48 | 0.93 | 1.6 | 0.09 | 0.82 |
| 10b2.pda.txt.after | OK | 20 | 2 | 2 | 1 |  |  |  |  |  |  |  |
| 11a1.pda.txt.before | OK | 21 | 1 | 1 | 1 | 0.45 | 0.65 | 0.1 | 0.84 | 1.23 | 0.14 | 0.76 |
| 11a1.pda.txt.after | OK | 21 | 1 | 2 | 1 |  |  |  |  |  |  |  |
| 11a2.pda.txt.before | OK | 21 | 2 | 1 | 1 | 1.22 | 1.8 | 0.38 | 0.84 | 1.23 | 0.14 | 0.76 |
| 11a2.pda.txt.after | OK | 21 | 2 | 2 | 1 |  |  |  |  |  |  |  |
| 11b1.pda.txt.before | OK | 22 | 1 | 1 | 1 | 1.23 | 2.36 | 0.36 | 1.03 | 1.92 | 0.27 | 0.8 |
| 11b1.pda.txt.after | OK | 22 | 1 | 2 | 1 |  |  |  |  |  |  |  |
| 11b2.pda.txt.before | OK | 22 | 2 | 1 | 1 | 0.83 | 1.48 | 0.18 | 1.03 | 1.92 | 0.27 | 0.8 |
| 11b2.pda.txt.after | OK | 22 | 2 | 2 | 1 |  |  |  |  |  |  |  |
| 12a1.pda.txt.before | OK | 23 | 1 | 1 | 1 | 0.64 | 1.2 | 1 | 0.85 | 1.51 | 0.06 | -0.11 |
| 12a1.pda.txt.after | OK | 23 | 1 | 2 | 1 |  |  |  |  |  |  |  |
| 12a2.pda.txt.before | OK | 23 | 2 | 1 | 1 | 1.05 | 1.82 | 0.88 | 0.85 | 1.51 | 0.06 | -0.11 |
| 12a2.pda.txt.after | OK | 23 | 2 | 2 | 1 |  |  |  |  |  |  |  |
| 12b1.pda.txt.before | OK | 24 | 1 | 1 | 1 | 0.58 | 1.09 | 0.63 | 0.87 | 1.64 | 0.07 | -0.07 |
| 12b1.pda.txt.after | OK | 24 | 1 | 2 | 1 |  |  |  |  |  |  |  |
| 12b2.pda.txt.before | OK | 24 | 2 | 1 | 1 | 1.16 | 2.2 | 0.76 | 0.87 | 1.64 | 0.07 | -0.07 |
| 12b2.pda.txt.after | OK | 24 | 2 | 2 | 1 |  |  |  |  |  |  |  |
| 13a1.pda.txt.before | OK | 25 | 1 | 1 | 1 | 0.09 | 0.79 | 0.17 | 0.02 | 1.02 | 0.21 | 0.51 |
| 13a1.pda.txt.after | OK | 25 | 1 | 2 | 1 |  |  |  |  |  |  |  |
| 13a3.pda.txt.before | OK | 25 | 2 | 1 | 1 | 0.06 | 1.24 | 0.25 | 0.02 | 1.02 | 0.21 | 0.51 |
| 13a3.pda.txt.after | OK | 25 | 2 | 2 | 1 |  |  |  |  |  |  |  |
| 13b1.pda.txt.before | OK | 26 | 1 | 1 | 1 | 0.42 | 0.91 | 0.14 | 0.58 | 1.13 | 0.33 | 0.67 |
| 13b1.pda.txt.after | OK | 26 | 1 | 2 | 1 |  |  |  |  |  |  |  |
| 13b2.pda.txt.before | OK | 26 | 2 | 1 | 1 | 0.74 | 1.36 | 0.51 | 0.58 | 1.13 | 0.33 | 0.67 |
| 13b2.pda.txt.after | OK | 26 | 2 | 2 | 1 |  |  |  |  |  |  |  |
| 14a1.pda.txt.before | OK | 27 | 1 | 1 | 1 | 0.3 | 0.74 | 0.38 | 0.12 | 0.83 | 0.43 | 0.48 |
| 14a1.pda.txt.after | OK | 27 | 1 | 2 | 1 |  |  |  |  |  |  |  |
| 14a3.pda.txt.before | OK | 27 | 2 | 1 | 1 | 0.06 | 0.93 | 0.49 | 0.12 | 0.83 | 0.43 | 0.48 |
| 14a3.pda.txt.after | OK | 27 | 2 | 2 | 1 |  |  |  |  |  |  |  |
| 14b1.pda.txt.before | OK | 28 | 1 | 1 | 1 | 0.51 | 1 | 0.2 | 0.7 | 1.24 | 0.3 | 0.11 |
| 14b1.pda.txt.after | OK | 28 | 1 | 2 | 1 |  |  |  |  |  |  |  |
| 14b3.pda.txt.before | OK | 28 | 2 | 1 | 1 | 0.9 | 1.48 | 0.8 | 0.7 | 1.24 | 0.3 | 0.11 |
| 14b3.pda.txt.after | OK | 28 | 2 | 2 | 1 |  |  |  |  |  |  |  |
| 15a1.pda.txt.before | OK | 29 | 1 | 1 | 1 | 0.21 | 0.97 | 0.32 | 0.29 | 1.06 | 0.01 | 0.79 |
| 15a1.pda.txt.after | OK | 29 | 1 | 2 | 1 |  |  |  |  |  |  |  |
| 15a2.pda.txt.before | OK | 29 | 2 | 1 | 1 | 0.37 | 1.16 | 0.35 | 0.29 | 1.06 | 0.01 | 0.79 |
| 15a2.pda.txt.after | OK | 29 | 2 | 2 | 1 |  |  |  |  |  |  |  |
| 15b1.pda.txt.before | OK | 30 | 1 | 1 | 1 | 0.41 | 0.87 | 0.07 | 0.44 | 1.01 | 0.05 | 0.79 |
| 15b1.pda.txt.after | OK | 30 | 1 | 2 | 1 |  |  |  |  |  |  |  |
| 15b2.pda.txt.before | OK | 30 | 2 | 1 | 1 | 0.48 | 1.14 | 0.16 | 0.44 | 1.01 | 0.05 | 0.79 |
| 15b2.pda.txt.after | OK | 30 | 2 | 2 | 1 |  |  |  |  |  |  |  |
| 16a1.pda.txt.before | OK | 31 | 1 | 1 | 1 | 1.03 | 1.24 | 0.64 | 0.72 | 1.14 | 0.28 | 0.62 |
| 16a1.pda.txt.after | OK | 31 | 1 | 2 | 1 |  |  |  |  |  |  |  |
| 16a3.pda.txt.before | OK | 31 | 2 | 1 | 1 | 0.4 | 1.03 | 0.09 | 0.72 | 1.14 | 0.28 | 0.62 |
| 16a3.pda.txt.after | OK | 31 | 2 | 2 | 1 |  |  |  |  |  |  |  |
| 16b1.pda.txt.before | OK | 32 | 1 | 1 | 1 | 0.42 | 0.98 | 0.07 | 0.54 | 1.11 | 0.28 | 0.72 |
| 16b1.pda.txt.after | OK | 32 | 1 | 2 | 1 |  |  |  |  |  |  |  |
| 16b3.pda.txt.before | OK | 32 | 2 | 1 | 1 | 0.65 | 1.23 | 0.63 | 0.54 | 1.11 | 0.28 | 0.72 |
| 16b3.pda.txt.after | OK | 32 | 2 | 2 | 1 |  |  |  |  |  |  |  |
| 17a1.pda.txt.before | OK | 33 | 1 | 1 | 1 | 0.5 | 0.91 | 0.16 | 0.63 | 1.16 | 0.35 | 0.72 |
| 17a1.pda.txt.after | OK | 33 | 1 | 2 | 1 |  |  |  |  |  |  |  |
| 17a2.pda.txt.before | OK | 33 | 2 | 1 | 1 | 0.76 | 1.41 | 0.54 | 0.63 | 1.16 | 0.35 | 0.72 |
| 17a2.pda.txt.after | OK | 33 | 2 | 2 | 1 |  |  |  |  |  |  |  |
| 17b1.pda.txt.before | OK | 34 | 1 | 1 | 1 | 0.5 | 0.91 | 0.08 | 0.49 | 1.13 | 0.35 | 0.89 |
| 17b1.pda.txt.after | OK | 34 | 1 | 2 | 1 |  |  |  |  |  |  |  |
| 17b2.pda.txt.before | OK | 34 | 2 | 1 | 1 | 0.48 | 1.36 | 0.63 | 0.49 | 1.13 | 0.35 | 0.89 |
| 17b2.pda.txt.after | OK | 34 | 2 | 2 | 1 |  |  |  |  |  |  |  |
| 18a1.pda.txt.before | OK | 35 | 1 | 1 | 1 | 0.64 | 1.62 | 0.25 | 0.72 | 1.61 | 0.49 | 0.77 |
| 18a1.pda.txt.after | OK | 35 | 1 | 2 | 1 |  |  |  |  |  |  |  |
| 18a2.pda.txt.before | OK | 35 | 2 | 1 | 1 | 0.79 | 1.6 | 0.73 | 0.72 | 1.61 | 0.49 | 0.77 |
| 18a2.pda.txt.after | OK | 35 | 2 | 2 | 1 |  |  |  |  |  |  |  |
| 18b1.pda.txt.before | OK | 36 | 1 | 1 | 1 | 0.66 | 1.58 | 0.1 | 0.52 | 1.55 | 0.23 | 0.85 |
| 18b1.pda.txt.after | OK | 36 | 1 | 2 | 1 |  |  |  |  |  |  |  |
| 18b2.pda.txt.before | OK | 36 | 2 | 1 | 1 | 0.37 | 1.51 | 0.56 | 0.52 | 1.55 | 0.23 | 0.85 |
| 18b2.pda.txt.after | OK | 36 | 2 | 2 | 1 |  |  |  |  |  |  |  |
| 19a1.pda.txt.before | OK | 37 | 1 | 1 | 1 | 0.79 | 1.35 | 0.21 | 0.91 | 1.63 | 0.55 | 0.86 |
| 19a1.pda.txt.after | OK | 37 | 1 | 2 | 1 |  |  |  |  |  |  |  |
| 19a2.pda.txt.before | OK | 37 | 2 | 1 | 1 | 1.03 | 1.91 | 1.32 | 0.91 | 1.63 | 0.55 | 0.86 |
| 19a2.pda.txt.after | OK | 37 | 2 | 2 | 1 |  |  |  |  |  |  |  |
| 19b1.pda.txt.before | OK | 38 | 1 | 1 | 1 | 0.74 | 1.36 | 0.04 | 0.93 | 1.6 | 0.56 | 0.82 |
| 19b1.pda.txt.after | OK | 38 | 1 | 2 | 1 |  |  |  |  |  |  |  |
| 19b2.pda.txt.before | OK | 38 | 2 | 1 | 1 | 1.12 | 1.85 | 1.17 | 0.93 | 1.6 | 0.56 | 0.82 |
| 19b2.pda.txt.after | OK | 38 | 2 | 2 | 1 |  |  |  |  |  |  |  |
| 20a1.pda.txt.before | OK | 39 | 1 | 1 | 1 | 0.93 | 1.39 | 0.14 | 1.2 | 1.84 | 0.59 | 0.82 |
| 20a1.pda.txt.after | OK | 39 | 1 | 2 | 1 |  |  |  |  |  |  |  |
| 20a2.pda.txt.before | OK | 39 | 2 | 1 | 1 | 1.47 | 2.29 | 1.04 | 1.2 | 1.84 | 0.59 | 0.82 |
| 20a2.pda.txt.after | OK | 39 | 2 | 2 | 1 |  |  |  |  |  |  |  |
| 20b1.pda.txt.before | OK | 40 | 1 | 1 | 1 | 0.72 | 1.12 | 0.25 | 0.67 | 1.49 | 0.06 | 0.86 |
| 20b1.pda.txt.after | OK | 40 | 1 | 2 | 1 |  |  |  |  |  |  |  |
| 20b2.pda.txt.before | OK | 40 | 2 | 1 | 1 | 0.63 | 1.86 | 0.36 | 0.67 | 1.49 | 0.06 | 0.86 |
| 20b2.pda.txt.after | OK | 40 | 2 | 2 | 1 |  |  |  |  |  |  |  |
| 21a3.pda.txt.before | OK | 41 | 1 | 1 | 1 | 0.51 | 1.5 | 0.3 | 0.92 | 1.84 | 0.48 | 0.79 |
| 21a3.pda.txt.after | OK | 41 | 1 | 2 | 1 |  |  |  |  |  |  |  |
| 21a2.pda.txt.before | OK | 41 | 2 | 1 | 1 | 1.34 | 2.17 | 0.66 | 0.92 | 1.84 | 0.48 | 0.79 |
| 21a2.pda.txt.after | OK | 41 | 2 | 2 | 1 |  |  |  |  |  |  |  |
| 21b3.pda.txt.before | OK | 42 | 1 | 1 | 1 | 1.44 | 2.07 | 0.57 | 1.35 | 1.97 | 0.67 | 0.77 |
| 21b3.pda.txt.after | OK | 42 | 1 | 2 | 1 |  |  |  |  |  |  |  |
| 21b2.pda.txt.before | OK | 42 | 2 | 1 | 1 | 1.26 | 1.86 | 0.77 | 1.35 | 1.97 | 0.67 | 0.77 |
| 21b2.pda.txt.after | OK | 42 | 2 | 2 | 1 |  |  |  |  |  |  |  |
| 22a3.pda.txt.before | OK | 43 | 1 | 1 | 1 | 0.82 | 1.5 | 0.08 | 0.84 | 1.41 | 0.54 | 0.5 |
| 22a3.pda.txt.after | OK | 43 | 1 | 2 | 1 |  |  |  |  |  |  |  |
| 22a2.pda.txt.before | OK | 43 | 2 | 1 | 1 | 0.86 | 1.31 | 1.01 | 0.84 | 1.41 | 0.54 | 0.5 |
| 22a2.pda.txt.after | OK | 43 | 2 | 2 | 1 |  |  |  |  |  |  |  |
| 22b3.pda.txt.before | OK | 44 | 1 | 1 | 1 | 0.13 | 1.13 | 0.55 | 0.66 | 1.33 | 0.7 | 0.22 |
| 22b3.pda.txt.after | OK | 44 | 1 | 2 | 1 |  |  |  |  |  |  |  |
| 22b2.pda.txt.before | OK | 44 | 2 | 1 | 1 | 1.19 | 1.54 | 0.85 | 0.66 | 1.33 | 0.7 | 0.22 |
| 22b2.pda.txt.after | OK | 44 | 2 | 2 | 1 |  |  |  |  |  |  |  |
| 23a1.pda.txt.before | OK | 45 | 1 | 1 | 1 | 0.08 | 0.66 | 0.36 | 0.59 | 1.22 | 0.29 | -0.07 |
| 23a1.pda.txt.after | OK | 45 | 1 | 2 | 1 |  |  |  |  |  |  |  |
| 23a2.pda.txt.before | OK | 45 | 2 | 1 | 1 | 1.25 | 1.78 | 0.93 | 0.59 | 1.22 | 0.29 | -0.07 |
| 23a2.pda.txt.after | OK | 45 | 2 | 2 | 1 |  |  |  |  |  |  |  |
| 23b3.pda.txt.before | OK | 46 | 1 | 1 | 1 | 0.13 | 1.54 | 0.07 | 0.59 | 1.54 | 0.26 | 0.6 |
| 23b3.pda.txt.after | OK | 46 | 1 | 2 | 1 |  |  |  |  |  |  |  |
| 23b2.pda.txt.before | OK | 46 | 2 | 1 | 1 | 1.04 | 1.54 | 0.44 | 0.59 | 1.54 | 0.26 | 0.6 |
| 23b2.pda.txt.after | OK | 46 | 2 | 2 | 1 |  |  |  |  |  |  |  |
| 24a3.pda.txt.before | OK | 47 | 1 | 1 | 1 | 0.48 | 1.01 | 0.76 | 0.75 | 1.24 | 0.73 | 0.7 |
| 24a3.pda.txt.after | OK | 47 | 1 | 2 | 1 |  |  |  |  |  |  |  |
| 24a2.pda.txt.before | OK | 47 | 2 | 1 | 1 | 1.03 | 1.46 | 0.7 | 0.75 | 1.24 | 0.73 | 0.7 |
| 24a2.pda.txt.after | OK | 47 | 2 | 2 | 1 |  |  |  |  |  |  |  |
| 24b3.pda.txt.before | OK | 48 | 1 | 1 | 1 | 1.03 | NA | 0.48 | 0.96 | 1.43 | 0.55 | 0.81 |
| 24b3.pda.txt.after | OK | 48 | 1 | 2 | 1 |  |  |  |  |  |  |  |
| 24b2.pda.txt.before | OK | 48 | 2 | 1 | 1 | 0.89 | 1.43 | 0.62 | 0.96 | 1.43 | 0.55 | 0.81 |
| 24b2.pda.txt.after | OK | 48 | 2 | 2 | 1 |  |  |  |  |  |  |  |
| 25a3.pda.txt.before | OK | 49 | 1 | 1 | 1 | 0.61 | 1.41 | 0.05 | 0.9 | 2.1 | 0.51 | 0.34 |
| 25a3.pda.txt.after | OK | 49 | 1 | 2 | 1 |  |  |  |  |  |  |  |
| 25a2.pda.txt.before | OK | 49 | 2 | 1 | 1 | 1.2 | 2.78 | 0.98 | 0.9 | 2.1 | 0.51 | 0.34 |
| 25a2.pda.txt.after | OK | 49 | 2 | 2 | 1 |  |  |  |  |  |  |  |
| 25b3.pda.txt.before | OK | 50 | 1 | 1 | 1 | 1.36 | 1.91 | 1.07 | 1.14 | 2.32 | 0.88 | 0.76 |
| 25b3.pda.txt.after | OK | 50 | 1 | 2 | 1 |  |  |  |  |  |  |  |
| 25b2.pda.txt.before | OK | 50 | 2 | 1 | 1 | 0.91 | 2.72 | 0.69 | 1.14 | 2.32 | 0.88 | 0.76 |
| 25b2.pda.txt.after | OK | 50 | 2 | 2 | 1 |  |  |  |  |  |  |  |
| 26a3.pda.txt.before | OK | 51 | 1 | 1 | 1 | 0.49 | 1.68 | 0.59 | 0.79 | 2.15 | 0.65 | 0.26 |
| 26a3.pda.txt.after | OK | 51 | 1 | 2 | 1 |  |  |  |  |  |  |  |
| 26a2.pda.txt.before | OK | 51 | 2 | 1 | 1 | 1.09 | 2.62 | 0.71 | 0.79 | 2.15 | 0.65 | 0.26 |
| 26a2.pda.txt.after | OK | 51 | 2 | 2 | 1 |  |  |  |  |  |  |  |
| 26b3.pda.txt.before | OK | 52 | 1 | 1 | 1 | 1.18 | 1.68 | 1.16 | 1.11 | 2.1 | 0.64 | 0.63 |
| 26b3.pda.txt.after | OK | 52 | 1 | 2 | 1 |  |  |  |  |  |  |  |
| 26b2.pda.txt.before | OK | 52 | 2 | 1 | 1 | 1.04 | 2.52 | 0.12 | 1.11 | 2.1 | 0.64 | 0.63 |
| 26b2.pda.txt.after | OK | 52 | 2 | 2 | 1 |  |  |  |  |  |  |  |
| 27a3.pda.txt.before | OK | 53 | 1 | 1 | 1 | 0.43 | 1.66 | 0.13 | 0.2 | 1.12 | 0.25 | 0.26 |
| 27a3.pda.txt.after | OK | 53 | 1 | 2 | 1 |  |  |  |  |  |  |  |
| 27a2.pda.txt.before | OK | 53 | 2 | 1 | 1 | 0.83 | 0.57 | 0.64 | 0.2 | 1.12 | 0.25 | 0.26 |
| 27a2.pda.txt.after | OK | 53 | 2 | 2 | 1 |  |  |  |  |  |  |  |
| 27b1.pda.txt.before | OK | 54 | 1 | 1 | 1 | 0.53 | 1.27 | 0.41 | 0.58 | 1.92 | 0.14 | 0.39 |
| 27b1.pda.txt.after | OK | 54 | 1 | 2 | 1 |  |  |  |  |  |  |  |
| 27b2.pda.txt.before | OK | 54 | 2 | 1 | 1 | 0.64 | 2.57 | 0.7 | 0.58 | 1.92 | 0.14 | 0.39 |
| 27b2.pda.txt.after | OK | 54 | 2 | 2 | 1 |  |  |  |  |  |  |  |
| 28a1.pda.txt.before | OK | 55 | 1 | 1 | 1 | 0.41 | 0.97 | 0.11 | 0.63 | 1.77 | 0.34 | 0.75 |
| 28a1.pda.txt.after | OK | 55 | 1 | 2 | 1 |  |  |  |  |  |  |  |
| 28a2.pda.txt.before | OK | 55 | 2 | 1 | 1 | 0.85 | 2.58 | 0.78 | 0.63 | 1.77 | 0.34 | 0.75 |
| 28a2.pda.txt.after | OK | 55 | 2 | 2 | 1 |  |  |  |  |  |  |  |
| 28b3.pda.txt.before | OK | 56 | 1 | 1 | 1 | 0.24 | 1.44 | 0.15 | 1.03 | 2.27 | 0.91 | 0.4 |
| 28b3.pda.txt.after | OK | 56 | 1 | 2 | 1 |  |  |  |  |  |  |  |
| 28b2.pda.txt.before | OK | 56 | 2 | 1 | 1 | 1.83 | 3.1 | 1.97 | 1.03 | 2.27 | 0.91 | 0.4 |
| 28b2.pda.txt.after | OK | 56 | 2 | 2 | 1 |  |  |  |  |  |  |  |
| 29a3.pda.txt.before | OK | 57 | 1 | 1 | 1 | 0.83 | 1.78 | 0.86 | 0.69 | 1.91 | 0.42 | 0.18 |
| 29a3.pda.txt.after | OK | 57 | 1 | 2 | 1 |  |  |  |  |  |  |  |
| 29a2.pda.txt.before | OK | 57 | 2 | 1 | 1 | 0.54 | 2.04 | 0.01 | 0.69 | 1.91 | 0.42 | 0.18 |
| 29a2.pda.txt.after | OK | 57 | 2 | 2 | 1 |  |  |  |  |  |  |  |
| 29b3.pda.txt.before | OK | 58 | 1 | 1 | 1 | 0.3 | 1.43 | 0.21 | 0.72 | 2.19 | 0.77 | 0.69 |
| 29b3.pda.txt.after | OK | 58 | 1 | 2 | 1 |  |  |  |  |  |  |  |
| 29b2.pda.txt.before | OK | 58 | 2 | 1 | 1 | 1.14 | 2.95 | 1.33 | 0.72 | 2.19 | 0.77 | 0.69 |
| 29b2.pda.txt.after | OK | 58 | 2 | 2 | 1 |  |  |  |  |  |  |  |
| 30a3.pda.txt.before | OK | 59 | 1 | 1 | 1 | 0.61 | 1.34 | 0.64 | 0.75 | 1.76 | 0.74 | 0.69 |
| 30a3.pda.txt.after | OK | 59 | 1 | 2 | 1 |  |  |  |  |  |  |  |
| 30a2.pda.txt.before | OK | 59 | 2 | 1 | 1 | 0.9 | 2.18 | 0.84 | 0.75 | 1.76 | 0.74 | 0.69 |
| 30a2.pda.txt.after | OK | 59 | 2 | 2 | 1 |  |  |  |  |  |  |  |
| 30b3.pda.txt.before | OK | 60 | 1 | 1 | 1 | 1.03 | 1.76 | 0.54 | 0.98 | 2.14 | 1.11 | 0.32 |
| 30b3.pda.txt.after | OK | 60 | 1 | 2 | 1 |  |  |  |  |  |  |  |
| 30b2.pda.txt.before | OK | 60 | 2 | 1 | 1 | 0.94 | 2.52 | 1.69 | 0.98 | 2.14 | 1.11 | 0.32 |
| 30b2.pda.txt.after | OK | 60 | 2 | 2 | 1 |  |  |  |  |  |  |  |
| 31a3.pda.txt.before | OK | 61 | 1 | 1 | 1 | 0.39 | 0.88 | 0.82 | 0.6 | 1.47 | 0.59 | 0.57 |
| 31a3.pda.txt.after | OK | 61 | 1 | 2 | 1 |  |  |  |  |  |  |  |
| 31a2.pda.txt.before | OK | 61 | 2 | 1 | 1 | 0.82 | 2.07 | 0.36 | 0.6 | 1.47 | 0.59 | 0.57 |
| 31a2.pda.txt.after | OK | 61 | 2 | 2 | 1 |  |  |  |  |  |  |  |
| 31b3.pda.txt.before | OK | 62 | 1 | 1 | 1 | 0.62 | 2.58 | 0.15 | 0.64 | 2.28 | 0.23 | 0.73 |
| 31b3.pda.txt.after | OK | 62 | 1 | 2 | 1 |  |  |  |  |  |  |  |
| 31b2.pda.txt.before | OK | 62 | 2 | 1 | 1 | 0.67 | 1.99 | 0.62 | 0.64 | 2.28 | 0.23 | 0.73 |
| 31b2.pda.txt.after | OK | 62 | 2 | 2 | 1 |  |  |  |  |  |  |  |
| 32a3.pda.txt.before | OK | 63 | 1 | 1 | 1 | 0.38 | 1.02 | 0.29 | 0.36 | 1.16 | 0.14 | 0.69 |
| 32a3.pda.txt.after | OK | 63 | 1 | 2 | 1 |  |  |  |  |  |  |  |
| 32a2.pda.txt.before | OK | 63 | 2 | 1 | 1 | 0.33 | 1.3 | 0 | 0.36 | 1.16 | 0.14 | 0.69 |
| 32a2.pda.txt.after | OK | 63 | 2 | 2 | 1 |  |  |  |  |  |  |  |
| 32b3.pda.txt.before | OK | 64 | 1 | 1 | 1 | 0.76 | 1.25 | 0.32 | 0.78 | 1.59 | 0.25 | 0.56 |
| 32b3.pda.txt.after | OK | 64 | 1 | 2 | 1 |  |  |  |  |  |  |  |
| 32b2.pda.txt.before | OK | 64 | 2 | 1 | 1 | 0.8 | 1.92 | 0.18 | 0.78 | 1.59 | 0.25 | 0.56 |
| 32b2.pda.txt.after | OK | 64 | 2 | 2 | 1 |  |  |  |  |  |  |  |
| 33a3.pda.txt.before | OK | 65 | 1 | 1 | 1 | 0.62 | 1.1 | 0.33 | 0.31 | 1.36 | 0.19 | 0.75 |
| 33a3.pda.txt.after | OK | 65 | 1 | 2 | 1 |  |  |  |  |  |  |  |
| 33a2.pda.txt.before | OK | 65 | 2 | 1 | 1 | 0.01 | 1.63 | 0.04 | 0.31 | 1.36 | 0.19 | 0.75 |
| 33a2.pda.txt.after | OK | 65 | 2 | 2 | 1 |  |  |  |  |  |  |  |
| 33b1.pda.txt.before | OK | 66 | 1 | 1 | 1 | 1.05 | 1.69 | 0.29 | 0.63 | 1.31 | 0.11 | 0.55 |
| 33b1.pda.txt.after | OK | 66 | 1 | 2 | 1 |  |  |  |  |  |  |  |
| 33b3.pda.txt.before | OK | 66 | 2 | 1 | 1 | 0.22 | 0.93 | 0.06 | 0.63 | 1.31 | 0.11 | 0.55 |
| 33b3.pda.txt.after | OK | 66 | 2 | 2 | 1 |  |  |  |  |  |  |  |
| 34a1.pda.txt.before | OK | 67 | 1 | 1 | 1 | 0.97 | 1.05 | 0.13 | 0.68 | 1.1 | 0.19 | 0.83 |
| 34a1.pda.txt.after | OK | 67 | 1 | 2 | 1 |  |  |  |  |  |  |  |
| 34a2.pda.txt.before | OK | 67 | 2 | 1 | 1 | 0.4 | 1.15 | 0.25 | 0.68 | 1.1 | 0.19 | 0.83 |
| 34a2.pda.txt.after | OK | 67 | 2 | 2 | 1 |  |  |  |  |  |  |  |
| 34b1.pda.txt.before | OK | 68 | 1 | 1 | 1 | 0.76 | 1.37 | 0.17 | 0.43 | 1.2 | 0.07 | 0.73 |
| 34b1.pda.txt.after | OK | 68 | 1 | 2 | 1 |  |  |  |  |  |  |  |
| 34b2.pda.txt.before | OK | 68 | 2 | 1 | 1 | 0.1 | 1.02 | 0.04 | 0.43 | 1.2 | 0.07 | 0.73 |
| 34b2.pda.txt.after | OK | 68 | 2 | 2 | 1 |  |  |  |  |  |  |  |
| 35a1.pda.txt.before | OK | 69 | 1 | 1 | 1 | 0.84 | 1.23 | 0.09 | 0.76 | 1.36 | 0.16 | 0.82 |
| 35a1.pda.txt.after | OK | 69 | 1 | 2 | 1 |  |  |  |  |  |  |  |
| 35a2.pda.txt.before | OK | 69 | 2 | 1 | 1 | 0.68 | 1.49 | 0.23 | 0.76 | 1.36 | 0.16 | 0.82 |
| 35a2.pda.txt.after | OK | 69 | 2 | 2 | 1 |  |  |  |  |  |  |  |
| 35b1.pda.txt.before | OK | 70 | 1 | 1 | 1 | 0.72 | 1.53 | 0.04 | 0.84 | 1.6 | 0.04 | 0.83 |
| 35b1.pda.txt.after | OK | 70 | 1 | 2 | 1 |  |  |  |  |  |  |  |
| 35b2.pda.txt.before | OK | 70 | 2 | 1 | 1 | 0.96 | 1.68 | 0.11 | 0.84 | 1.6 | 0.04 | 0.83 |
| 35b2.pda.txt.after | OK | 70 | 2 | 2 | 1 |  |  |  |  |  |  |  |
| 36a3.pda.txt.before | OK | 71 | 1 | 1 | 1 | 0.95 | 0.86 | 0.77 | 0.83 | 1.2 | 0.53 | 0.55 |
| 36a3.pda.txt.after | OK | 71 | 1 | 2 | 1 |  |  |  |  |  |  |  |
| 36a2.pda.txt.before | OK | 71 | 2 | 1 | 1 | 0.71 | 1.54 | 0.28 | 0.83 | 1.2 | 0.53 | 0.55 |
| 36a2.pda.txt.after | OK | 71 | 2 | 2 | 1 |  |  |  |  |  |  |  |
| 36b1.pda.txt.before | OK | 72 | 1 | 1 | 1 | 1.01 | 1.77 | 0.51 | 0.58 | 1.57 | 0.16 | 0.74 |
| 36b1.pda.txt.after | OK | 72 | 1 | 2 | 1 |  |  |  |  |  |  |  |
| 36b2.pda.txt.before | OK | 72 | 2 | 1 | 1 | 0.14 | 1.37 | 0.19 | 0.58 | 1.57 | 0.16 | 0.74 |
| 36b2.pda.txt.after | OK | 72 | 2 | 2 | 1 |  |  |  |  |  |  |  |
| 37a1.pda.txt.before | OK | 73 | 1 | 1 | 1 | 1.34 | 1.62 | 0.6 | 1.07 | 1.57 | 0.76 | 0.82 |
| 37a1.pda.txt.after | OK | 73 | 1 | 2 | 1 |  |  |  |  |  |  |  |
| 37a2.pda.txt.before | OK | 73 | 2 | 1 | 1 | 0.8 | 1.51 | 0.91 | 1.07 | 1.57 | 0.76 | 0.82 |
| 37a2.pda.txt.after | OK | 73 | 2 | 2 | 1 |  |  |  |  |  |  |  |
| 37b3.pda.txt.before | OK | 74 | 1 | 1 | 1 | 0.06 | 1.24 | 0.15 | 0.27 | 1.46 | 0.2 | 0.58 |
| 37b3.pda.txt.after | OK | 74 | 1 | 2 | 1 |  |  |  |  |  |  |  |
| 37b2.pda.txt.before | OK | 74 | 2 | 1 | 1 | 0.61 | 1.68 | 0.24 | 0.27 | 1.46 | 0.2 | 0.58 |
| 37b2.pda.txt.after | OK | 74 | 2 | 2 | 1 |  |  |  |  |  |  |  |
| 38a1.pda.txt.before | OK | 75 | 1 | 1 | 1 | 0.95 | 1.62 | 0.12 | 0.73 | 1.49 | 0.23 | 0.78 |
| 38a1.pda.txt.after | OK | 75 | 1 | 2 | 1 |  |  |  |  |  |  |  |
| 38a2.pda.txt.before | OK | 75 | 2 | 1 | 1 | 0.51 | 1.35 | 0.33 | 0.73 | 1.49 | 0.23 | 0.78 |
| 38a2.pda.txt.after | OK | 75 | 2 | 2 | 1 |  |  |  |  |  |  |  |
| 38b1.pda.txt.before | OK | 76 | 1 | 1 | 1 | 0.71 | 1.35 | 0.09 | 0.82 | 1.62 | 0.26 | 0.84 |
| 38b1.pda.txt.after | OK | 76 | 1 | 2 | 1 |  |  |  |  |  |  |  |
| 38b2.pda.txt.before | OK | 76 | 2 | 1 | 1 | 0.93 | 1.9 | 0.6 | 0.82 | 1.62 | 0.26 | 0.84 |
| 38b2.pda.txt.after | OK | 76 | 2 | 2 | 1 |  |  |  |  |  |  |  |
| 39a1.pda.txt.before | OK | 77 | 1 | 1 | 1 | 1.24 | 1.74 | 0.64 | 1 | 1.66 | 0.79 | 0.78 |
| 39a1.pda.txt.after | OK | 77 | 1 | 2 | 1 |  |  |  |  |  |  |  |
| 39a2.pda.txt.before | OK | 77 | 2 | 1 | 1 | 0.76 | 1.59 | 0.94 | 1 | 1.66 | 0.79 | 0.78 |
| 39a2.pda.txt.after | OK | 77 | 2 | 2 | 1 |  |  |  |  |  |  |  |
| 39b1.pda.txt.before | OK | 78 | 1 | 1 | 1 | 0.77 | 1.57 | 0.08 | 0.79 | 1.59 | 0.46 | 0.79 |
| 39b1.pda.txt.after | OK | 78 | 1 | 2 | 1 |  |  |  |  |  |  |  |
| 39b2.pda.txt.before | OK | 78 | 2 | 1 | 1 | 0.81 | 1.62 | 0.83 | 0.79 | 1.59 | 0.46 | 0.79 |
| 39b2.pda.txt.after | OK | 78 | 2 | 2 | 1 |  |  |  |  |  |  |  |
| 40a1.pda.txt.before | OK | 79 | 1 | 1 | 1 | 0.78 | 1.46 | 0.18 | 0.84 | 1.46 | 0.34 | 0.68 |
| 40a1.pda.txt.after | OK | 79 | 1 | 2 | 1 |  |  |  |  |  |  |  |
| 40a2.pda.txt.before | OK | 79 | 2 | 1 | 1 | 0.9 | 1.45 | 0.49 | 0.84 | 1.46 | 0.34 | 0.68 |
| 40a2.pda.txt.after | OK | 79 | 2 | 2 | 1 |  |  |  |  |  |  |  |
| 40b1.pda.txt.before | OK | 80 | 1 | 1 | 1 | 1.2 | 1.82 | 0.52 | 1.06 | 1.85 | 0.72 | 0.75 |
| 40b1.pda.txt.after | OK | 80 | 1 | 2 | 1 |  |  |  |  |  |  |  |
| 40b2.pda.txt.before | OK | 80 | 2 | 1 | 1 | 0.92 | 1.89 | 0.93 | 1.06 | 1.85 | 0.72 | 0.75 |
| 40b2.pda.txt.after | OK | 80 | 2 | 2 | 1 |  |  |  |  |  |  |  |
| 41a1.pda.txt.before | OK | 81 | 1 | 1 | 1 | 0.92 | 1.62 | 0.57 | 0.85 | 1.72 | 0.69 | 0.83 |
| 41a1.pda.txt.after | OK | 81 | 1 | 2 | 1 |  |  |  |  |  |  |  |
| 41a2.pda.txt.before | OK | 81 | 2 | 1 | 1 | 0.77 | 1.83 | 0.81 | 0.85 | 1.72 | 0.69 | 0.83 |
| 41a2.pda.txt.after | OK | 81 | 2 | 2 | 1 |  |  |  |  |  |  |  |
| 41b1.pda.txt.before | OK | 82 | 1 | 1 | 1 | 0.99 | 1.61 | 0.29 | 0.68 | 1.57 | 0.46 | 0.79 |
| 41b1.pda.txt.after | OK | 82 | 1 | 2 | 1 |  |  |  |  |  |  |  |
| 41b2.pda.txt.before | OK | 82 | 2 | 1 | 1 | 0.37 | 1.53 | 0.63 | 0.68 | 1.57 | 0.46 | 0.79 |
| 41b2.pda.txt.after | OK | 82 | 2 | 2 | 1 |  |  |  |  |  |  |  |
| 42a1.pda.txt.before | OK | 83 | 1 | 1 | 1 | 0.71 | 1.5 | 0.46 | 0.99 | 1.52 | 0.55 | 0.29 |
| 42a1.pda.txt.after | OK | 83 | 1 | 2 | 1 |  |  |  |  |  |  |  |
| 42a2.pda.txt.before | OK | 83 | 2 | 1 | 1 | 1.27 | 1.53 | 0.65 | 0.99 | 1.52 | 0.55 | 0.29 |
| 42a2.pda.txt.after | OK | 83 | 2 | 2 | 1 |  |  |  |  |  |  |  |
| 42b1.pda.txt.before | OK | 84 | 1 | 1 | 1 | 0.61 | 1.53 | 0.32 | 0.3 | 1.19 | 0 | 0.24 |
| 42b1.pda.txt.after | OK | 84 | 1 | 2 | 1 |  |  |  |  |  |  |  |
| 42b3.pda.txt.before | OK | 84 | 2 | 1 | 1 | 0.01 | 0.84 | 0.31 | 0.3 | 1.19 | 0 | 0.24 |
| 42b3.pda.txt.after | OK | 84 | 2 | 2 | 1 |  |  |  |  |  |  |  |
| 43a1.pda.txt.before | OK | 85 | 1 | 1 | 1 | 1.22 | 1.48 | 0.35 | 1.1 | 1.61 | 0.77 | 0.79 |
| 43a1.pda.txt.after | OK | 85 | 1 | 2 | 1 |  |  |  |  |  |  |  |
| 43a2.pda.txt.before | OK | 85 | 2 | 1 | 1 | 0.99 | 1.74 | 1.19 | 1.1 | 1.61 | 0.77 | 0.79 |
| 43a2.pda.txt.after | OK | 85 | 2 | 2 | 1 |  |  |  |  |  |  |  |
| 43b1.pda.txt.before | OK | 86 | 1 | 1 | 1 | 0.5 | 1.56 | 0.48 | 0.64 | 1.75 | 0.72 | 0.71 |
| 43b1.pda.txt.after | OK | 86 | 1 | 2 | 1 |  |  |  |  |  |  |  |
| 43b2.pda.txt.before | OK | 86 | 2 | 1 | 1 | 0.79 | 1.93 | 0.96 | 0.64 | 1.75 | 0.72 | 0.71 |
| 43b2.pda.txt.after | OK | 86 | 2 | 2 | 1 |  |  |  |  |  |  |  |
| 44a3.pda.txt.before | OK | 87 | 1 | 1 | 1 | 0.19 | 0.9 | 0.03 | 0.65 | 1.28 | 0.5 | 0.39 |
| 44a3.pda.txt.after | OK | 87 | 1 | 2 | 1 |  |  |  |  |  |  |  |
| 44a2.pda.txt.before | OK | 87 | 2 | 1 | 1 | 1.11 | 1.67 | 1.03 | 0.65 | 1.28 | 0.5 | 0.39 |
| 44a2.pda.txt.after | OK | 87 | 2 | 2 | 1 |  |  |  |  |  |  |  |
| 44b1.pda.txt.before | OK | 88 | 1 | 1 | 1 | 0.78 | 1.46 | 0.17 | 0.8 | 1.71 | 0.34 | 0.67 |
| 44b1.pda.txt.after | OK | 88 | 1 | 2 | 1 |  |  |  |  |  |  |  |
| 44b2.pda.txt.before | OK | 88 | 2 | 1 | 1 | 0.83 | 1.96 | 0.51 | 0.8 | 1.71 | 0.34 | 0.67 |
| 44b2.pda.txt.after | OK | 88 | 2 | 2 | 1 |  |  |  |  |  |  |  |
| 45a1.pda.txt.before | OK | 89 | 1 | 1 | 1 | 0.5 | 1.3 | 0.03 | 0.85 | 1.98 | 0.8 | 0.72 |
| 45a1.pda.txt.after | OK | 89 | 1 | 2 | 1 |  |  |  |  |  |  |  |
| 45a2.pda.txt.before | OK | 89 | 2 | 1 | 1 | 1.2 | 2.66 | 1.63 | 0.85 | 1.98 | 0.8 | 0.72 |
| 45a2.pda.txt.after | OK | 89 | 2 | 2 | 1 |  |  |  |  |  |  |  |
| 45b3.pda.txt.before | OK | 90 | 1 | 1 | 1 | 0.38 | 1.08 | 0.34 | 0.77 | 1.65 | 0.94 | 0.59 |
| 45b3.pda.txt.after | OK | 90 | 1 | 2 | 1 |  |  |  |  |  |  |  |
| 45b2.pda.txt.before | OK | 90 | 2 | 1 | 1 | 1.15 | 2.23 | 1.53 | 0.77 | 1.65 | 0.94 | 0.59 |
| 45b2.pda.txt.after | OK | 90 | 2 | 2 | 1 |  |  |  |  |  |  |  |
| 46a1.pda.txt.before | OK | 91 | 1 | 1 | 1 | 0.54 | 1.27 | 0.32 | 0.89 | 1.6 | 0.62 | 0.77 |
| 46a1.pda.txt.after | OK | 91 | 1 | 2 | 1 |  |  |  |  |  |  |  |
| 46a2.pda.txt.before | OK | 91 | 2 | 1 | 1 | 1.24 | 1.92 | 0.92 | 0.89 | 1.6 | 0.62 | 0.77 |
| 46a2.pda.txt.after | OK | 91 | 2 | 2 | 1 |  |  |  |  |  |  |  |
| 46b1.pda.txt.before | OK | 92 | 1 | 1 | 1 | 1.18 | 1.72 | 0.57 | 1.29 | 1.94 | 0.77 | 0.66 |
| 46b1.pda.txt.after | OK | 92 | 1 | 2 | 1 |  |  |  |  |  |  |  |
| 46b2.pda.txt.before | OK | 92 | 2 | 1 | 1 | 1.4 | 2.16 | 0.97 | 1.29 | 1.94 | 0.77 | 0.66 |
| 46b2.pda.txt.after | OK | 92 | 2 | 2 | 1 |  |  |  |  |  |  |  |
| 47a1.pda.txt.before | OK | 93 | 1 | 1 | 1 | 1.61 | 1.68 | 0.36 | 1.37 | 1.93 | 0.47 | 0.8 |
| 47a1.pda.txt.after | OK | 93 | 1 | 2 | 1 |  |  |  |  |  |  |  |
| 47a2.pda.txt.before | OK | 93 | 2 | 1 | 1 | 1.12 | 2.18 | 0.58 | 1.37 | 1.93 | 0.47 | 0.8 |
| 47a2.pda.txt.after | OK | 93 | 2 | 2 | 1 |  |  |  |  |  |  |  |
| 47b1.pda.txt.before | OK | 94 | 1 | 1 | 1 | 0.6 | 1.42 | 0.01 | 0.71 | 1.67 | 0.36 | 0.78 |
| 47b1.pda.txt.after | OK | 94 | 1 | 2 | 1 |  |  |  |  |  |  |  |
| 47b2.pda.txt.before | OK | 94 | 2 | 1 | 1 | 0.82 | 1.93 | 0.71 | 0.71 | 1.67 | 0.36 | 0.78 |
| 47b2.pda.txt.after | OK | 94 | 2 | 2 | 1 |  |  |  |  |  |  |  |
| 48a3.pda.txt.before | OK | 95 | 1 | 1 | 1 | 0.12 | 0.95 | 0.22 | 0.63 | 1.38 | 0.45 | 0.84 |
| 48a3.pda.txt.after | OK | 95 | 1 | 2 | 1 |  |  |  |  |  |  |  |
| 48a2.pda.txt.before | OK | 95 | 2 | 1 | 1 | 1.14 | 1.82 | 0.68 | 0.63 | 1.38 | 0.45 | 0.84 |
| 48a2.pda.txt.after | OK | 95 | 2 | 2 | 1 |  |  |  |  |  |  |  |
| 48b1.pda.txt.before | OK | 96 | 1 | 1 | 1 | 0.7 | 1.15 | 0.53 | 0.48 | 1.22 | 0.12 | -0.1 |
| 48b1.pda.txt.after | OK | 96 | 1 | 2 | 1 |  |  |  |  |  |  |  |
| 48b2.pda.txt.before | OK | 96 | 2 | 1 | 1 | 0.26 | 1.28 | 0.29 | 0.48 | 1.22 | 0.12 | -0.1 |
| 48b2.pda.txt.after | OK | 96 | 2 | 2 | 1 |  |  |  |  |  |  |  |
| 49a3.pda.txt.before | OK | 97 | 1 | 1 | 1 | 0.08 | 1.79 | 0.24 | 0.08 | 1.79 | 0.24 | 1 |
| 49a3.pda.txt.after | OK | 97 | 1 | 2 | 1 |  |  |  |  |  |  |  |
| 49a32.pda.txt.before | OK | 97 | 2 | 1 | 1 | 0.08 | 1.79 | 0.24 | 0.08 | 1.79 | 0.24 | 1 |
| 49a32.pda.txt.after | OK | 97 | 2 | 2 | 1 |  |  |  |  |  |  |  |
| 49b3.pda.txt.before | OK | 98 | 1 | 1 | 1 | 1.33 | 2.81 | 0.38 | 1.33 | 2.81 | 0.38 | 1 |
| 49b3.pda.txt.after | OK | 98 | 1 | 2 | 1 |  |  |  |  |  |  |  |
| 49b32.pda.txt.before | OK | 98 | 2 | 1 | 1 | 1.33 | 2.81 | 0.38 | 1.33 | 2.81 | 0.38 | 1 |
| 49b32.pda.txt.after | OK | 98 | 2 | 2 | 1 |  |  |  |  |  |  |  |
| 50a3.pda.txt.before | OK | 99 | 1 | 1 | 1 | 0.36 | 2.53 | 0.62 | 0.54 | 2.34 | 0.15 | 0.81 |
| 50a3.pda.txt.after | OK | 99 | 1 | 2 | 1 |  |  |  |  |  |  |  |
| 50a2.pda.txt.before | OK | 99 | 2 | 1 | 1 | 0.73 | 2.15 | 0.31 | 0.54 | 2.34 | 0.15 | 0.81 |
| 50a2.pda.txt.after | OK | 99 | 2 | 2 | 1 |  |  |  |  |  |  |  |
| 50b1.pda.txt.before | OK | 100 | 1 | 1 | 1 | 1.02 | 1.52 | 0.35 | 0.69 | 2.05 | 0.08 | 0.1 |
| 50b1.pda.txt.after | OK | 100 | 1 | 2 | 1 |  |  |  |  |  |  |  |
| 50b3.pda.txt.before | OK | 100 | 2 | 1 | 1 | 0.36 | 2.59 | 0.19 | 0.69 | 2.05 | 0.08 | 0.1 |
| 50b3.pda.txt.after | OK | 100 | 2 | 2 | 1 |  |  |  |  |  |  |  |
| 51a1.pda.txt.before | OK | 101 | 1 | 1 | 1 | 1.31 | 1.59 | 0.08 | 1.14 | 1.71 | 0.12 | 0.73 |
| 51a1.pda.txt.after | OK | 101 | 1 | 2 | 1 |  |  |  |  |  |  |  |
| 51a2.pda.txt.before | OK | 101 | 2 | 1 | 1 | 0.97 | 1.84 | 0.17 | 1.14 | 1.71 | 0.12 | 0.73 |
| 51a2.pda.txt.after | OK | 101 | 2 | 2 | 1 |  |  |  |  |  |  |  |
| 51b3.pda.txt.before | OK | 102 | 1 | 1 | 1 | 0.63 | 2.61 | 0.05 | 1.11 | 2.54 | 0.34 | 0.79 |
| 51b3.pda.txt.after | OK | 102 | 1 | 2 | 1 |  |  |  |  |  |  |  |
| 51b2.pda.txt.before | OK | 102 | 2 | 1 | 1 | 1.6 | 2.47 | 0.74 | 1.11 | 2.54 | 0.34 | 0.79 |
| 51b2.pda.txt.after | OK | 102 | 2 | 2 | 1 |  |  |  |  |  |  |  |
| 52a1.pda.txt.before | OK | 103 | 1 | 1 | 1 | 0.36 | 1.43 | 0.19 | 0.39 | 1.52 | 0.08 | 0.55 |
| 52a1.pda.txt.after | OK | 103 | 1 | 2 | 1 |  |  |  |  |  |  |  |
| 52a2.pda.txt.before | OK | 103 | 2 | 1 | 1 | 0.43 | 1.61 | 0.03 | 0.39 | 1.52 | 0.08 | 0.55 |
| 52a2.pda.txt.after | OK | 103 | 2 | 2 | 1 |  |  |  |  |  |  |  |
| 52b1.pda.txt.before | OK | 104 | 1 | 1 | 1 | 0.72 | 1.39 | 0.09 | 1.07 | 2.25 | 0.35 | 0.74 |
| 52b1.pda.txt.after | OK | 104 | 1 | 2 | 1 |  |  |  |  |  |  |  |
| 52b3.pda.txt.before | OK | 104 | 2 | 1 | 1 | 1.42 | 3.12 | 0.61 | 1.07 | 2.25 | 0.35 | 0.74 |
| 52b3.pda.txt.after | OK | 104 | 2 | 2 | 1 |  |  |  |  |  |  |  |
| 53a3.pda.txt.before | OK | 105 | 1 | 1 | 1 | 0.44 | 1.53 | 0.85 | 0.17 | 1.57 | 0.31 | 0.54 |
| 53a3.pda.txt.after | OK | 105 | 1 | 2 | 1 |  |  |  |  |  |  |  |
| 53a2.pda.txt.before | OK | 105 | 2 | 1 | 1 | 0.78 | 1.61 | 0.23 | 0.17 | 1.57 | 0.31 | 0.54 |
| 53a2.pda.txt.after | OK | 105 | 2 | 2 | 1 |  |  |  |  |  |  |  |
| 53b1.pda.txt.before | OK | 106 | 1 | 1 | 1 | 1.9 | 2.44 | 1.02 | 0.19 | 1.72 | 0.22 | 0.66 |
| 53b1.pda.txt.after | OK | 106 | 1 | 2 | 1 |  |  |  |  |  |  |  |
| 53b3.pda.txt.before | OK | 106 | 2 | 1 | 1 | 1.52 | 0.99 | 0.58 | 0.19 | 1.72 | 0.22 | 0.66 |
| 53b3.pda.txt.after | OK | 106 | 2 | 2 | 1 |  |  |  |  |  |  |  |
| 54a1.pda.txt.before | OK | 107 | 1 | 1 | 1 | 0.39 | 1.24 | 0.02 | 0.46 | 2.2 | 0.2 | 0.6 |
| 54a1.pda.txt.after | OK | 107 | 1 | 2 | 1 |  |  |  |  |  |  |  |
| 54a3.pda.txt.before | OK | 107 | 2 | 1 | 1 | 0.53 | 3.16 | 0.42 | 0.46 | 2.2 | 0.2 | 0.6 |
| 54a3.pda.txt.after | OK | 107 | 2 | 2 | 1 |  |  |  |  |  |  |  |
| 54b1.pda.txt.before | OK | 108 | 1 | 1 | 1 | 1.16 | 1.79 | 0.65 | 1.23 | 1.83 | 0.77 | 0.79 |
| 54b1.pda.txt.after | OK | 108 | 1 | 2 | 1 |  |  |  |  |  |  |  |
| 54b2.pda.txt.before | OK | 108 | 2 | 1 | 1 | 1.3 | 1.86 | 0.9 | 1.23 | 1.83 | 0.77 | 0.79 |
| 54b2.pda.txt.after | OK | 108 | 2 | 2 | 1 |  |  |  |  |  |  |  |
| 55a1.pda.txt.before | OK | 109 | 1 | 1 | 1 | 1.03 | 2.01 | 0.58 | 0.84 | 1.62 | 0.12 | 0.61 |
| 55a1.pda.txt.after | OK | 109 | 1 | 2 | 1 |  |  |  |  |  |  |  |
| 55a2.pda.txt.before | OK | 109 | 2 | 1 | 1 | 0.65 | 1.24 | 0.34 | 0.84 | 1.62 | 0.12 | 0.61 |
| 55a2.pda.txt.after | OK | 109 | 2 | 2 | 1 |  |  |  |  |  |  |  |
| 55b1.pda.txt.before | OK | 110 | 1 | 1 | 1 | 1.18 | 1.64 | 0.65 | 0.83 | 2.11 | 0.56 | 0.37 |
| 55b1.pda.txt.after | OK | 110 | 1 | 2 | 1 |  |  |  |  |  |  |  |
| 55b3.pda.txt.before | OK | 110 | 2 | 1 | 1 | 0.48 | 2.58 | 0.46 | 0.83 | 2.11 | 0.56 | 0.37 |
| 55b3.pda.txt.after | OK | 110 | 2 | 2 | 1 |  |  |  |  |  |  |  |
| 56a1.pda.txt.before | OK | 111 | 1 | 1 | 1 | 1.12 | 1.95 | 0.79 | 1.1 | 1.64 | 0.41 | 0.92 |
| 56a1.pda.txt.after | OK | 111 | 1 | 2 | 1 |  |  |  |  |  |  |  |
| 56a2.pda.txt.before | OK | 111 | 2 | 1 | 1 | 1.09 | 1.33 | 0.04 | 1.1 | 1.64 | 0.41 | 0.92 |
| 56a2.pda.txt.after | OK | 111 | 2 | 2 | 1 |  |  |  |  |  |  |  |
| 56b1.pda.txt.before | OK | 112 | 1 | 1 | 1 | 0.82 | 1.65 | 0.53 | 0.83 | 1.4 | 0.24 | 0.86 |
| 56b1.pda.txt.after | OK | 112 | 1 | 2 | 1 |  |  |  |  |  |  |  |
| 56b2.pda.txt.before | OK | 112 | 2 | 1 | 1 | 0.85 | 1.15 | 0.05 | 0.83 | 1.4 | 0.24 | 0.86 |
| 56b2.pda.txt.after | OK | 112 | 2 | 2 | 1 |  |  |  |  |  |  |  |
| 57a1.pda.txt.before | OK | 113 | 1 | 1 | 1 | 0.65 | 1.64 | 0.52 | 0.71 | 2.39 | 0.38 | 0.49 |
| 57a1.pda.txt.after | OK | 113 | 1 | 2 | 1 |  |  |  |  |  |  |  |
| 57a3.pda.txt.before | OK | 113 | 2 | 1 | 1 | 0.77 | 3.14 | 0.24 | 0.71 | 2.39 | 0.38 | 0.49 |
| 57a3.pda.txt.after | OK | 113 | 2 | 2 | 1 |  |  |  |  |  |  |  |
| 57b1.pda.txt.before | OK | 114 | 1 | 1 | 1 | 1.04 | 1.55 | 0.21 | 0.58 | 1.43 | 0.12 | 0.49 |
| 57b1.pda.txt.after | OK | 114 | 1 | 2 | 1 |  |  |  |  |  |  |  |
| 57b32.pda.txt.before | OK | 114 | 2 | 1 | 1 | 0.12 | 1.31 | 0.03 | 0.58 | 1.43 | 0.12 | 0.49 |
| 57b32.pda.txt.after | OK | 114 | 2 | 2 | 1 |  |  |  |  |  |  |  |
| 58a1.pda.txt.before | OK | 115 | 1 | 1 | 1 | 0.97 | 1.39 | 0.13 | 1.32 | 1.66 | 0.63 | 0.74 |
| 58a1.pda.txt.after | OK | 115 | 1 | 2 | 1 |  |  |  |  |  |  |  |
| 58a2.pda.txt.before | OK | 115 | 2 | 1 | 1 | 1.68 | 1.93 | 1.13 | 1.32 | 1.66 | 0.63 | 0.74 |
| 58a2.pda.txt.after | OK | 115 | 2 | 2 | 1 |  |  |  |  |  |  |  |
| 58b2.pda.txt.before | OK | 116 | 2 | 1 | 1 | 1.67 | 1.98 | 0.91 | 1.67 | 1.98 | 0.91 | NA |
| 58b2.pda.txt.after | OK | 116 | 2 | 2 | 1 |  |  |  |  |  |  |  |
| 59a2.pda.txt.before | OK | 117 | 2 | 1 | 1 | 0.64 | 1.21 | 0.38 | 0.64 | 1.21 | 0.38 | NA |
| 59a2.pda.txt.after | OK | 117 | 2 | 2 | 1 |  |  |  |  |  |  |  |
| 59b1.pda.txt.before | OK | 118 | 1 | 1 | 1 | 0.83 | 1.54 | 0.29 | 1.3 | 1.89 | 0.63 | 0.67 |
| 59b1.pda.txt.after | OK | 118 | 1 | 2 | 1 |  |  |  |  |  |  |  |
| 59b2.pda.txt.before | OK | 118 | 2 | 1 | 1 | 1.77 | 2.23 | 0.96 | 1.3 | 1.89 | 0.63 | 0.67 |
| 59b2.pda.txt.after | OK | 118 | 2 | 2 | 1 |  |  |  |  |  |  |  |
| 60a1.pda.txt.before | OK | 119 | 1 | 1 | 1 | 0.34 | 1.17 | 0.47 | 0.88 | 1.46 | 0.48 | 0.69 |
| 60a1.pda.txt.after | OK | 119 | 1 | 2 | 1 |  |  |  |  |  |  |  |
| 60a2.pda.txt.before | OK | 119 | 2 | 1 | 1 | 1.42 | 1.75 | 0.5 | 0.88 | 1.46 | 0.48 | 0.69 |
| 60a2.pda.txt.after | OK | 119 | 2 | 2 | 1 |  |  |  |  |  |  |  |
| 60b1.pda.txt.before | OK | 120 | 1 | 1 | 1 | 1.08 | 1.7 | 0.56 | 0.98 | 1.6 | 0.45 | 0.7 |
| 60b1.pda.txt.after | OK | 120 | 1 | 2 | 1 |  |  |  |  |  |  |  |
| 60b2.pda.txt.before | OK | 120 | 2 | 1 | 1 | 0.87 | 1.49 | 0.34 | 0.98 | 1.6 | 0.45 | 0.7 |
| 60b2.pda.txt.after | OK | 120 | 2 | 2 | 1 |  |  |  |  |  |  |  |
| 61a3.pda.txt.before | OK | 121 | 1 | 1 | 1 | 1.06 | 0.98 | 0.58 | 0.54 | 1.07 | 0.05 | 0.59 |
| 61a3.pda.txt.after | OK | 121 | 1 | 2 | 1 |  |  |  |  |  |  |  |
| 61a2.pda.txt.before | OK | 121 | 2 | 1 | 1 | 0.03 | 1.17 | 0.68 | 0.54 | 1.07 | 0.05 | 0.59 |
| 61a2.pda.txt.after | OK | 121 | 2 | 2 | 1 |  |  |  |  |  |  |  |
| 61b1.pda.txt.before | OK | 122 | 1 | 1 | 1 | 1.41 | 1.76 | 0.86 | 1.04 | 1.58 | 0.43 | 0.87 |
| 61b1.pda.txt.after | OK | 122 | 1 | 2 | 1 |  |  |  |  |  |  |  |
| 61b2.pda.txt.before | OK | 122 | 2 | 1 | 1 | 0.67 | 1.4 | 0.01 | 1.04 | 1.58 | 0.43 | 0.87 |
| 61b2.pda.txt.after | OK | 122 | 2 | 2 | 1 |  |  |  |  |  |  |  |
| 62a3.pda.txt.before | OK | 123 | 1 | 1 | 1 | 0.54 | 1.19 | 0.07 | 0.83 | 1.5 | 0.53 | 0.77 |
| 62a3.pda.txt.after | OK | 123 | 1 | 2 | 1 |  |  |  |  |  |  |  |
| 62a2.pda.txt.before | OK | 123 | 2 | 1 | 1 | 1.11 | 1.8 | 0.98 | 0.83 | 1.5 | 0.53 | 0.77 |
| 62a2.pda.txt.after | OK | 123 | 2 | 2 | 1 |  |  |  |  |  |  |  |
| 62b3.pda.txt.before | OK | 124 | 1 | 1 | 1 | 0.37 | 1.28 | 0.11 | 0.7 | 1.6 | 0.42 | 0.5 |
| 62b3.pda.txt.after | OK | 124 | 1 | 2 | 1 |  |  |  |  |  |  |  |
| 62b2.pda.txt.before | OK | 124 | 2 | 1 | 1 | 1.03 | 1.93 | 0.72 | 0.7 | 1.6 | 0.42 | 0.5 |
| 62b2.pda.txt.after | OK | 124 | 2 | 2 | 1 |  |  |  |  |  |  |  |
| 63a1.pda.txt.before | OK | 125 | 1 | 1 | 1 | 1.71 | 2.16 | 1.18 | 1.03 | 1.76 | 0.84 | 0.76 |
| 63a1.pda.txt.after | OK | 125 | 1 | 2 | 1 |  |  |  |  |  |  |  |
| 63a2.pda.txt.before | OK | 125 | 2 | 1 | 1 | 0.35 | 1.37 | 0.5 | 1.03 | 1.76 | 0.84 | 0.76 |
| 63a2.pda.txt.after | OK | 125 | 2 | 2 | 1 |  |  |  |  |  |  |  |
| 63b32.pda.txt.before | OK | 126 | 1 | 1 | 1 | 0.32 | 0.84 | 0.5 | 0.35 | 0.97 | 0.3 | 0.51 |
| 63b32.pda.txt.after | OK | 126 | 1 | 2 | 1 |  |  |  |  |  |  |  |
| 63b3.pda.txt.before | OK | 126 | 2 | 1 | 1 | 0.37 | 1.1 | 0.1 | 0.35 | 0.97 | 0.3 | 0.51 |
| 63b3.pda.txt.after | OK | 126 | 2 | 2 | 1 |  |  |  |  |  |  |  |
| 64a1.pda.txt.before | OK | 127 | 1 | 1 | 1 | 1.02 | 1.77 | 0.23 | 0.73 | 1.51 | 0.29 | 0.52 |
| 64a1.pda.txt.after | OK | 127 | 1 | 2 | 1 |  |  |  |  |  |  |  |
| 64a3.pda.txt.before | OK | 127 | 2 | 1 | 1 | 0.44 | 1.24 | 0.35 | 0.73 | 1.51 | 0.29 | 0.52 |
| 64a3.pda.txt.after | OK | 127 | 2 | 2 | 1 |  |  |  |  |  |  |  |
| 64b1.pda.txt.before | OK | 128 | 1 | 1 | 1 | 0.87 | 1.29 | 0.6 | 0.98 | 1.64 | 0.21 | 0.68 |
| 64b1.pda.txt.after | OK | 128 | 1 | 2 | 1 |  |  |  |  |  |  |  |
| 64b2.pda.txt.before | OK | 128 | 2 | 1 | 1 | 1.09 | 1.99 | 1.01 | 0.98 | 1.64 | 0.21 | 0.68 |
| 64b2.pda.txt.after | OK | 128 | 2 | 2 | 1 |  |  |  |  |  |  |  |
| 65a1.pda.txt.before | OK | 129 | 1 | 1 | 1 | 1.08 | 1.94 | 0.12 | 0.86 | 1.7 | 0.23 | 0.79 |
| 65a1.pda.txt.after | OK | 129 | 1 | 2 | 1 |  |  |  |  |  |  |  |
| 65a2.pda.txt.before | OK | 129 | 2 | 1 | 1 | 0.64 | 1.45 | 0.34 | 0.86 | 1.7 | 0.23 | 0.79 |
| 65a2.pda.txt.after | OK | 129 | 2 | 2 | 1 |  |  |  |  |  |  |  |
| 65b1.pda.txt.before | OK | 130 | 1 | 1 | 1 | 1.84 | 2.22 | 0.55 | 1.41 | 2.05 | 0.66 | 0.67 |
| 65b1.pda.txt.after | OK | 130 | 1 | 2 | 1 |  |  |  |  |  |  |  |
| 65b2.pda.txt.before | OK | 130 | 2 | 1 | 1 | 0.97 | 1.88 | 0.77 | 1.41 | 2.05 | 0.66 | 0.67 |
| 65b2.pda.txt.after | OK | 130 | 2 | 2 | 1 |  |  |  |  |  |  |  |
| 66a1.pda.txt.before | OK | 131 | 1 | 1 | 1 | 1.09 | 1.35 | 0.45 | 0.98 | 1.57 | 0.02 | 0.85 |
| 66a1.pda.txt.after | OK | 131 | 1 | 2 | 1 |  |  |  |  |  |  |  |
| 66a2.pda.txt.before | OK | 131 | 2 | 1 | 1 | 0.88 | 1.78 | 0.49 | 0.98 | 1.57 | 0.02 | 0.85 |
| 66a2.pda.txt.after | OK | 131 | 2 | 2 | 1 |  |  |  |  |  |  |  |
| 66b32.pda.txt.before | OK | 132 | 1 | 1 | 1 | 0.41 | 1.11 | 0.09 | 0.51 | 1.14 | 0.21 | 0.63 |
| 66b32.pda.txt.after | OK | 132 | 1 | 2 | 1 |  |  |  |  |  |  |  |
| 66b3.pda.txt.before | OK | 132 | 2 | 1 | 1 | 0.61 | 1.17 | 0.32 | 0.51 | 1.14 | 0.21 | 0.63 |
| 66b3.pda.txt.after | OK | 132 | 2 | 2 | 1 |  |  |  |  |  |  |  |
| 67a1.pda.txt.before | OK | 133 | 1 | 1 | 1 | 0.77 | 1.56 | 0.64 | 1.07 | 1.79 | 0.23 | 0.02 |
| 67a1.pda.txt.after | OK | 133 | 1 | 2 | 1 |  |  |  |  |  |  |  |
| 67a2.pda.txt.before | OK | 133 | 2 | 1 | 1 | 1.38 | 2.03 | 1.1 | 1.07 | 1.79 | 0.23 | 0.02 |
| 67a2.pda.txt.after | OK | 133 | 2 | 2 | 1 |  |  |  |  |  |  |  |
| 67b3.pda.txt.before | OK | 134 | 1 | 1 | 1 | 0.09 | 0.95 | 0.28 | 0.41 | 1.3 | 0.4 | 0.63 |
| 67b3.pda.txt.after | OK | 134 | 1 | 2 | 1 |  |  |  |  |  |  |  |
| 67b2.pda.txt.before | OK | 134 | 2 | 1 | 1 | 0.91 | 1.66 | 1.08 | 0.41 | 1.3 | 0.4 | 0.63 |
| 67b2.pda.txt.after | OK | 134 | 2 | 2 | 1 |  |  |  |  |  |  |  |
| 68a1.pda.txt.before | OK | 135 | 1 | 1 | 1 | 0.8 | 1.56 | 0.08 | 0.72 | 2.01 | 0.1 | -0.27 |
| 68a1.pda.txt.after | OK | 135 | 1 | 2 | 1 |  |  |  |  |  |  |  |
| 68a3.pda.txt.before | OK | 135 | 2 | 1 | 1 | 0.64 | 2.46 | 0.13 | 0.72 | 2.01 | 0.1 | -0.27 |
| 68a3.pda.txt.after | OK | 135 | 2 | 2 | 1 |  |  |  |  |  |  |  |
| 68b1.pda.txt.before | OK | 136 | 1 | 1 | 1 | 1.57 | 2.02 | 0.15 | 1.12 | 1.88 | 0.27 | 0.07 |
| 68b1.pda.txt.after | OK | 136 | 1 | 2 | 1 |  |  |  |  |  |  |  |
| 68b2.pda.txt.before | OK | 136 | 2 | 1 | 1 | 0.68 | 1.73 | 0.39 | 1.12 | 1.88 | 0.27 | 0.07 |
| 68b2.pda.txt.after | OK | 136 | 2 | 2 | 1 |  |  |  |  |  |  |  |
| 69a1.pda.txt.before | OK | 137 | 1 | 1 | 1 | 1.11 | 1.75 | 0.03 | 0.61 | 1.49 | 0.04 | 0.8 |
| 69a1.pda.txt.after | OK | 137 | 1 | 2 | 1 |  |  |  |  |  |  |  |
| 69a2.pda.txt.before | OK | 137 | 2 | 1 | 1 | 0.1 | 1.23 | 0.11 | 0.61 | 1.49 | 0.04 | 0.8 |
| 69a2.pda.txt.after | OK | 137 | 2 | 2 | 1 |  |  |  |  |  |  |  |
| 69b1.pda.txt.before | OK | 138 | 1 | 1 | 1 | 1.25 | 1.72 | 0.38 | 0.85 | 1.54 | 0.36 | 0.67 |
| 69b1.pda.txt.after | OK | 138 | 1 | 2 | 1 |  |  |  |  |  |  |  |
| 69b2.pda.txt.before | OK | 138 | 2 | 1 | 1 | 0.45 | 1.36 | 0.34 | 0.85 | 1.54 | 0.36 | 0.67 |
| 69b2.pda.txt.after | OK | 138 | 2 | 2 | 1 |  |  |  |  |  |  |  |
| 70a1.pda.txt.before | OK | 139 | 1 | 1 | 1 | 1.27 | 2.06 | 1.3 | 1.11 | 2.03 | 0.96 | 0.87 |
| 70a1.pda.txt.after | OK | 139 | 1 | 2 | 1 |  |  |  |  |  |  |  |
| 70a2.pda.txt.before | OK | 139 | 2 | 1 | 1 | 0.95 | 2.01 | 0.61 | 1.11 | 2.03 | 0.96 | 0.87 |
| 70a2.pda.txt.after | OK | 139 | 2 | 2 | 1 |  |  |  |  |  |  |  |
| 70b1.pda.txt.before | OK | 140 | 1 | 1 | 1 | 0.83 | 1.52 | 0.71 | 1.05 | 1.74 | 0.85 | 0.69 |
| 70b1.pda.txt.after | OK | 140 | 1 | 2 | 1 |  |  |  |  |  |  |  |
| 70b2.pda.txt.before | OK | 140 | 2 | 1 | 1 | 1.27 | 1.97 | 0.99 | 1.05 | 1.74 | 0.85 | 0.69 |
| 70b2.pda.txt.after | OK | 140 | 2 | 2 | 1 |  |  |  |  |  |  |  |
| 71a1.pda.txt.before | OK | 141 | 1 | 1 | 1 | 1.34 | 2.08 | 0.82 | 0.7 | 2.4 | 0.07 | 0.59 |
| 71a1.pda.txt.after | OK | 141 | 1 | 2 | 1 |  |  |  |  |  |  |  |
| 71a3.pda.txt.before | OK | 141 | 2 | 1 | 1 | 0.05 | 2.72 | 0.95 | 0.7 | 2.4 | 0.07 | 0.59 |
| 71a3.pda.txt.after | OK | 141 | 2 | 2 | 1 |  |  |  |  |  |  |  |
| 71b1.pda.txt.before | OK | 142 | 1 | 1 | 1 | 1.11 | 1.88 | 0.78 | 1.03 | 1.9 | 0.74 | 0.73 |
| 71b1.pda.txt.after | OK | 142 | 1 | 2 | 1 |  |  |  |  |  |  |  |
| 71b2.pda.txt.before | OK | 142 | 2 | 1 | 1 | 0.96 | 1.92 | 0.7 | 1.03 | 1.9 | 0.74 | 0.73 |
| 71b2.pda.txt.after | OK | 142 | 2 | 2 | 1 |  |  |  |  |  |  |  |
| 72a1.pda.txt.before | OK | 143 | 1 | 1 | 1 | 0.43 | 1.68 | 0.26 | 0.35 | 1.62 | 0.26 | 0.61 |
| 72a1.pda.txt.after | OK | 143 | 1 | 2 | 1 |  |  |  |  |  |  |  |
| 72a2.pda.txt.before | OK | 143 | 2 | 1 | 1 | 0.27 | 1.56 | 0.25 | 0.35 | 1.62 | 0.26 | 0.61 |
| 72a2.pda.txt.after | OK | 143 | 2 | 2 | 1 |  |  |  |  |  |  |  |
| 72b1.pda.txt.before | OK | 144 | 1 | 1 | 1 | 0.78 | 2.02 | 0.63 | 0.93 | 2.01 | 0.66 | 0.62 |
| 72b1.pda.txt.after | OK | 144 | 1 | 2 | 1 |  |  |  |  |  |  |  |
| 72b2.pda.txt.before | OK | 144 | 2 | 1 | 1 | 1.09 | 2 | 0.68 | 0.93 | 2.01 | 0.66 | 0.62 |
| 72b2.pda.txt.after | OK | 144 | 2 | 2 | 1 |  |  |  |  |  |  |  |
| 73a1.pda.txt.before | OK | 145 | 1 | 1 | 1 | 1.14 | 2.21 | 1.09 | 0.7 | 1.81 | 0.69 | 0.51 |
| 73a1.pda.txt.after | OK | 145 | 1 | 2 | 1 |  |  |  |  |  |  |  |
| 73a2.pda.txt.before | OK | 145 | 2 | 1 | 1 | 0.27 | 1.4 | 0.29 | 0.7 | 1.81 | 0.69 | 0.51 |
| 73a2.pda.txt.after | OK | 145 | 2 | 2 | 1 |  |  |  |  |  |  |  |
| 73b1.pda.txt.before | OK | 146 | 1 | 1 | 1 | 0.49 | 1.65 | 0.47 | 0.32 | 1.91 | 0.03 | 0.62 |
| 73b1.pda.txt.after | OK | 146 | 1 | 2 | 1 |  |  |  |  |  |  |  |
| 73b3.pda.txt.before | OK | 146 | 2 | 1 | 1 | 0.14 | 2.18 | 0.53 | 0.32 | 1.91 | 0.03 | 0.62 |
| 73b3.pda.txt.after | OK | 146 | 2 | 2 | 1 |  |  |  |  |  |  |  |
| 74a1.pda.txt.before | OK | 147 | 1 | 1 | 1 | 1.45 | 2.23 | 1.31 | 1.15 | 2.31 | 0.82 | 0.47 |
| 74a1.pda.txt.after | OK | 147 | 1 | 2 | 1 |  |  |  |  |  |  |  |
| 74a3.pda.txt.before | OK | 147 | 2 | 1 | 1 | 0.85 | 2.38 | 0.34 | 1.15 | 2.31 | 0.82 | 0.47 |
| 74a3.pda.txt.after | OK | 147 | 2 | 2 | 1 |  |  |  |  |  |  |  |
| 74b1.pda.txt.before | OK | 148 | 1 | 1 | 1 | 0.95 | 1.92 | 1.28 | 0.82 | 1.52 | 1.07 | 0.71 |
| 74b1.pda.txt.after | OK | 148 | 1 | 2 | 1 |  |  |  |  |  |  |  |
| 74b2.pda.txt.before | OK | 148 | 2 | 1 | 1 | 0.69 | 1.13 | 0.85 | 0.82 | 1.52 | 1.07 | 0.71 |
| 74b2.pda.txt.after | OK | 148 | 2 | 2 | 1 |  |  |  |  |  |  |  |
| 75a1.pda.txt.before | OK | 149 | 1 | 1 | 1 | 0.74 | 2.37 | 1.59 | 0.65 | 1.93 | 0.84 | 0.77 |
| 75a1.pda.txt.after | OK | 149 | 1 | 2 | 1 |  |  |  |  |  |  |  |
| 75a2.pda.txt.before | OK | 149 | 2 | 1 | 1 | 0.55 | 1.5 | 0.09 | 0.65 | 1.93 | 0.84 | 0.77 |
| 75a2.pda.txt.after | OK | 149 | 2 | 2 | 1 |  |  |  |  |  |  |  |
| 75b1.pda.txt.before | OK | 150 | 1 | 1 | 1 | 0.12 | 1.63 | 1.57 | 0.37 | 1.64 | 1.04 | 0.76 |
| 75b1.pda.txt.after | OK | 150 | 1 | 2 | 1 |  |  |  |  |  |  |  |
| 75b2.pda.txt.before | OK | 150 | 2 | 1 | 1 | 0.63 | 1.65 | 0.52 | 0.37 | 1.64 | 1.04 | 0.76 |
| 75b2.pda.txt.after | OK | 150 | 2 | 2 | 1 |  |  |  |  |  |  |  |
| 76a1.pda.txt.before | OK | 151 | 1 | 1 | 1 | 1.35 | 2.13 | 0.38 | 1.03 | 1.96 | 0.45 | 0.67 |
| 76a1.pda.txt.after | OK | 151 | 1 | 2 | 1 |  |  |  |  |  |  |  |
| 76a2.pda.txt.before | OK | 151 | 2 | 1 | 1 | 0.72 | 1.78 | 0.52 | 1.03 | 1.96 | 0.45 | 0.67 |
| 76a2.pda.txt.after | OK | 151 | 2 | 2 | 1 |  |  |  |  |  |  |  |
| 76b1.pda.txt.before | OK | 152 | 1 | 1 | 1 | 0.79 | 1.77 | 0.32 | 0.92 | 1.86 | 0.51 | 0.66 |
| 76b1.pda.txt.after | OK | 152 | 1 | 2 | 1 |  |  |  |  |  |  |  |
| 76b2.pda.txt.before | OK | 152 | 2 | 1 | 1 | 1.06 | 1.95 | 0.71 | 0.92 | 1.86 | 0.51 | 0.66 |
| 76b2.pda.txt.after | OK | 152 | 2 | 2 | 1 |  |  |  |  |  |  |  |
| 77a1.pda.txt.before | OK | 153 | 1 | 1 | 1 | 1.09 | 1.45 | 0.61 | 0.72 | 1.55 | 0.36 | 0.48 |
| 77a1.pda.txt.after | OK | 153 | 1 | 2 | 1 |  |  |  |  |  |  |  |
| 77a3.pda.txt.before | OK | 153 | 2 | 1 | 1 | 0.35 | 1.66 | 1.32 | 0.72 | 1.55 | 0.36 | 0.48 |
| 77a3.pda.txt.after | OK | 153 | 2 | 2 | 1 |  |  |  |  |  |  |  |
| 77b1.pda.txt.before | OK | 154 | 1 | 1 | 1 | 0.7 | 1.51 | 0.74 | 0.63 | 1.74 | 0.71 | 0.11 |
| 77b1.pda.txt.after | OK | 154 | 1 | 2 | 1 |  |  |  |  |  |  |  |
| 77b2.pda.txt.before | OK | 154 | 2 | 1 | 1 | 0.55 | 1.97 | 0.69 | 0.63 | 1.74 | 0.71 | 0.11 |
| 77b2.pda.txt.after | OK | 154 | 2 | 2 | 1 |  |  |  |  |  |  |  |

  
  

|  | Channel 1 |
| --- | --- |
| Replicate 1 |  |
|  |  |
| Replicate 2 |  |
|  |  |
|  | Screen-wide image plot of the scored values |
| *Table of scored   and annotated probes* | enlarged version |

  

---

Sat Nov 10 17:43:05 2007
